# Space-time characterization of community noise and sound sources in Accra, Ghana

**DOI:** 10.1038/s41598-021-90454-6

**Published:** 2021-05-27

**Authors:** Sierra N. Clark, Abosede S. Alli, Ricky Nathvani, Allison Hughes, Majid Ezzati, Michael Brauer, Mireille B. Toledano, Jill Baumgartner, James E. Bennett, James Nimo, Josephine Bedford Moses, Solomon Baah, Samuel Agyei-Mensah, George Owusu, Briony Croft, Raphael E. Arku

**Affiliations:** 1 Department of Epidemiology and Biostatistics, School of Public Health, Imperial College London, London, UK; 2 MRC Centre for Environment and Health, School of Public Health, Imperial College London, London, UK; 3 Department of Environmental Health Sciences, School of Public Health and Health Sciences, University of Massachusetts, Amherst, MA USA; 4 Department of Physics, University of Ghana, Accra, Ghana; 5 Regional Institute for Population Studies, University of Ghana, Accra, Ghana; 6 Abdul Latif Jameel Institute for Disease and Emergency Analytics, Imperial College London, London, UK; 7 School of Population and Public Health, The University of British Columbia, Vancouver, Canada; 8 Mohn Centre for Children’s Health and Wellbeing, School of Public Health, Imperial College London, London, UK; 9 Institute for Health and Social Policy, McGill University, Montreal, Canada; 10 Department of Epidemiology, Biostatistics, and Occupational Health, McGill University, Montreal, Canada; 11 Department of Geography and Resource Development, University of Ghana, Accra, Ghana; 12 Institute of Statistical, Social & Economic Research, University of Ghana, Accra, Ghana; 13 SLR Consulting, Vancouver, Canada

**Keywords:** Risk factors, Environmental impact, Epidemiology

## Abstract

Urban noise pollution is an emerging public health concern in growing cities in sub-Saharan Africa (SSA), but the sound environment in SSA cities is understudied. We leveraged a large-scale measurement campaign to characterize the spatial and temporal patterns of measured sound levels and sound sources in Accra, Ghana. We measured sound levels and recorded audio clips at 146 representative locations, involving 7-days (136 locations) and 1-year measurements between 2019 and 2020. We calculated metrics of noise levels and intermittency and analyzed audio recordings using a pre-trained neural network to identify sources. Commercial, business, and industrial areas and areas near major roads had the highest median daily sound levels (LAeq_24hr_: 69 dBA and 72 dBA) and the lowest percentage of intermittent sound; the vice-versa was found for peri urban areas. Road-transport sounds dominated the overall sound environment but mixtures of other sound sources, including animals, human speech, and outdoor music, dominated in various locations and at different times. Environmental noise levels in Accra exceeded both international and national health-based guidelines. Detailed information on the acoustical environmental quality (including sound levels and types) in Accra may guide environmental policy formulation and evaluation to improve the health of urban residents.

## Introduction

Over half of the world’s population now lives in cities ^1^. Though urban living can remarkably improve livelihood and health through increased access to urban infrastructure, technology, and services, environmental exposures in cities can erode these health benefits and worsen the quality of life of urban residents ^2^. This is particularly the case in Sub-Saharan Africa (SSA), currently the world’s fastest urbanizing region ^1^. With increasing economic activities, transportation, and demand for energy, urban growth in SSA is marked by environmental pollution, including noise pollution ^3–6^. Epidemiologic studies conducted primarily in North America and Europe have linked exposure to road, rail, and aircraft traffic noise pollution to a range of health and well-being outcomes, such as sleep disturbance ^7^, annoyance ^8, 9^, impaired cognitive functioning ^10, 11^, and cardiovascular diseases ^12, 13^. Small-scale studies in SSA have shown that community noise levels at specific areas surpassed local guidelines ^3, 14–18^. However, large scale monitoring activities as seen in North America and Europe are lacking in SSA resulting in limited data to understand the distribution of noise pollution and sound sources in cities ^19–23^. Thus, there is a significant unmet need to undertake rigorous scientific research on community noise in SSA cities to support public health policy and regulatory decisions.

While community noise characterization has typically focused on measurement and/or models of average sound levels ^21, 22, 24^, effective management and prioritization of noise pollution control require a thorough understanding of spatial and temporal patterns in relation to sources. Particularly in SSA urban settings where there are potentially diverse sources of community sound, information about daily, weekly, and seasonal patterns will help identify appropriate interventions to mitigate noise and promote acoustic environments that can enhance health and well-being (e.g., nature sounds) ^25–28^. Accordingly, we combined sound level measurements with audio recordings to descriptively characterize the levels and diversity of sound sources over spatial and temporal strata in the West African city of Accra, Ghana, with a large-scale and comprehensive field campaign from combined weeklong (136 ‘rotating’ sites) and yearlong (10 ‘fixed’ sites) measurements. This work was nested within the “Pathways to Equitable Healthy Cities” study (http://equitablehealthycities.or.g/), which aims to identify and put into action equitable and healthy urban development and revitalization pathways in six cities on four continents.

### Study area

Our study was conducted in the Greater Accra Metropolitan Area (GAMA, ~5 million people), the most densely populated area in Ghana and the political, economic and administrative capital. Accounting for over a fifth of the urban population, this metropolitan region includes Accra Metropolitan Area (AMA) as its core (population: ~ 2 million), Tema Metropolitan Area to the east, and the expanding suburban municipalities to the north east and north west ^29^. As the host of the national capital, GAMA has attracted both public and private investments and become a hub for business, technology, and education ^30^. While these sectors drive urban economic growth, there also remain large inequalities in income, housing, and exposure to environmental pollutants ^31–33^.

## Results

### Spatial and temporal patterns of sound levels

**Figure 1:**
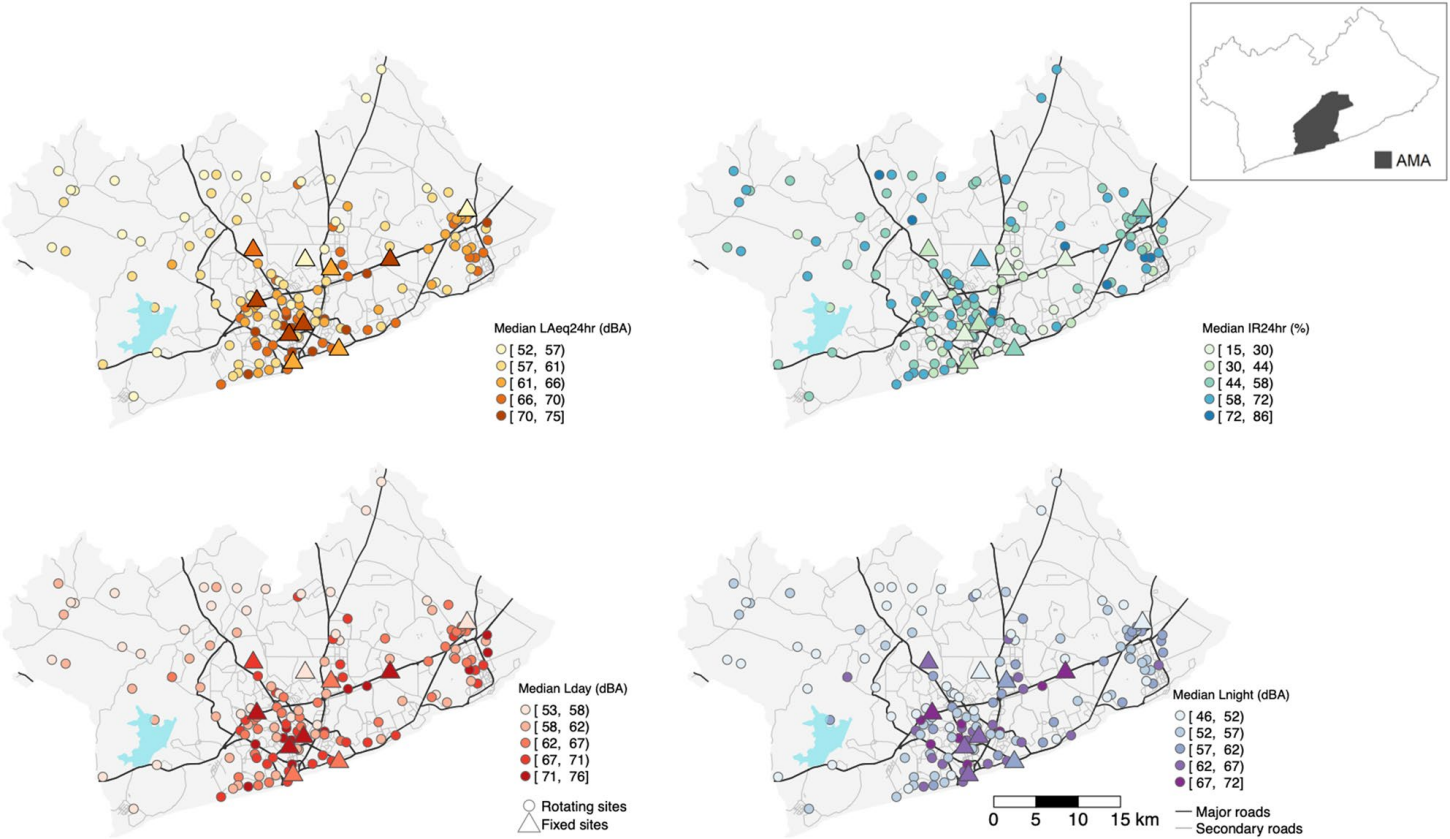
Median sound level and intermittency metrics for rotating and fixed site locations in the GAMA. For the color legend scales, a bracket means that the value in the range is included and a parenthesis means that the value in the range is not included. Road network is from OpenStreetMap and the Greater Accra Metropolitan Area (GAMA) and Accra Metropolitan Area (AMA) boundary from Ghana Statistical Service. A waterbody is depicted on the map in light blue. LAeq_24hr_: A-weighted equivalent continuous 24-h sound level; L_day_ and L_night_: A-weighted equivalent continuous sound level in the day and night-time; IR_24hr_: 24-h Intermittency Ratio.

The median daily A-weighted equivalent continuous sound level (LAeq_24hr_) across the GAMA was 62 dBA (interquartile range (IQR): 58, 67) and 66 dBA (IQR: 61, 70) in AMA (i.e., main urban centre) from measurements conducted at 136 locations for weeklong periods. There was variability in measured sound levels within and between land use categorizations, and the median value across land use categories were different (*p* value (*p*) < 0.01). Commercial, business, and industrial (CBI) areas had the highest median daily sound level (69 dBA, IQR: 66, 71) and peri-urban areas in the north, northwest and northeast of the GAMA the lowest (56 dBA, IQR: 54, 59) (Table 1; see Methods for description of land use classes). High-density residential areas had higher median daily sound levels compared with medium/low-density residential areas. In general, the difference in daily sound levels (LAeq_24hr_) at the 25th and 75th percentiles of the distribution within land use areas ranged from 5–6 dBA (Table 1). In the daytime, the variability was greatest among measurement sites in residential areas and lowest among CBI sites. In the night-time the variability was highest among high-density residential sites and lowest in peri-urban areas. Figure 1 shows the spatial patterns of sound levels mapped at the measurement site across the GAMA. The lowest daily sound levels were measured at sites in the outlying geographic areas of the GAMA and the highest at sites in the city-core (AMA), to the east near Tema and Ashaiman, and along major road networks. For the measurement sites along major roads (highways, motorways), the median day-time sound level (L_day_) was 74 dBA (IQR: 73, 75), and was also elevated at night (median 68 dBA (IQR: 67, 70)).

**Table 1:** Noise metrics in the Greater Accra Metropolitan Area and across land use areas.

Metrics and units	LAeq_24hr_ dBA	L_day_ dBA	L_night_ dBA	IR_24hr_ %	IR_day_ %	IR_night_ %
**Rotating sites**						
Greater Accra Metropolitan Area	62 (58, 67)	63 (59, 68)	55 (51, 61)	53 (37, 64)	46 (32, 59)	52 (35, 67)
Accra Metropolitan Area	66 (61, 70)	67 (62, 71)	58 (53, 64)	49 (31, 60)	40 (25, 53)	51 (39, 66)
Land use*						
Peri-urban background	56 (54, 59)	57 (55, 60)	50 (49, 53)	60 (51, 68)	58 (47, 67)	40 (20, 58)
Medium/low-density residential	61 (58, 64)	62 (59, 66)	54 (51, 57)	55 (42, 64)	48 (35, 59)	57 (38, 69)
High-density residential	67 (63, 69)	68 (64, 70)	60 (54, 64)	51 (35, 64)	43 (29, 57)	57 (38, 69)
Commercial, business, industrial	69 (66, 71)	70 (68, 72)	63 (60, 67)	36 (27, 54)	30 (18, 44)	50 (36, 65)
Fixed sites**	69 (63, 72)	70 (64, 73)	64 (58, 68)	33 (19, 49)	23 (11, 40)	41 (30, 54)

Data are expressed as medians and interquartile ranges (IQR). Each of the 136 rotating sites have 7-days of measurement and fixed sites have data spanning 12-months.

LAeq_24hr_: A-weighted equivalent continuous 24-h sound level; L_day_ and L_night_: A-weighted equivalent continuous sound level in the day and night-time; IR_24hr_: 24-h Intermittency Ratio; IR_day_ and IR_night_: Day- and night-time Intermittency Ratio.

*Medium/low-density residential are typically formal residential areas; high-density residential are typically informal residential areas and could be classified as shantytowns or slums; peri-urban background are the least built-up areas and typically have an abundance of forest, grass land, shrubs, barren land, and/or water; commercial, business and industrial areas are places which are dominated by commercial, business, industrial, and/or government activities (see full criteria in S8).

**10 fixed sites had 3 CBI areas along major motorways, 2 sites in high-density residential neighborhoods, 4 sites in medium/low-density residential areas and one at a peri-urban background location.

We found that the median Intermittency Ratio in the GAMA, which expresses the amount of intermittent sound in a defined period of time, was ~ 50% for both the day and night-time (Table 1), when a fixed cutoff of 3 dBA above the site and date specific day and night-time sound level was used. Median Intermittency Ratios were different between land use areas with *p* < 0.01, with the exception of nighttime in high-density and medium/low-density residential areas. Peri-urban areas had the highest median daily Intermittency Ratios (60%, IQR: 51, 68), while CBI areas had the lowest (36%, IQR: 27, 54). Additionally, sites along major roads had a low median Intermittency Ratio (7%, IQR: 5, 12) in the daytime and moderate-low in the night-time (31%, IQR: 27, 32); at night-time, background ambient noise decreases, and noise events can be more clearly identified. As expected, when higher fixed thresholds were used to calculate the daily Intermittency Ratios, the median levels were reduced (46% with + 4 dBA and 39% with + 5 dBA). Though the IR_24hr_ calculated with varying fixed thresholds was highly correlated (more information in the supplementary information S1).

**Figure 2:**
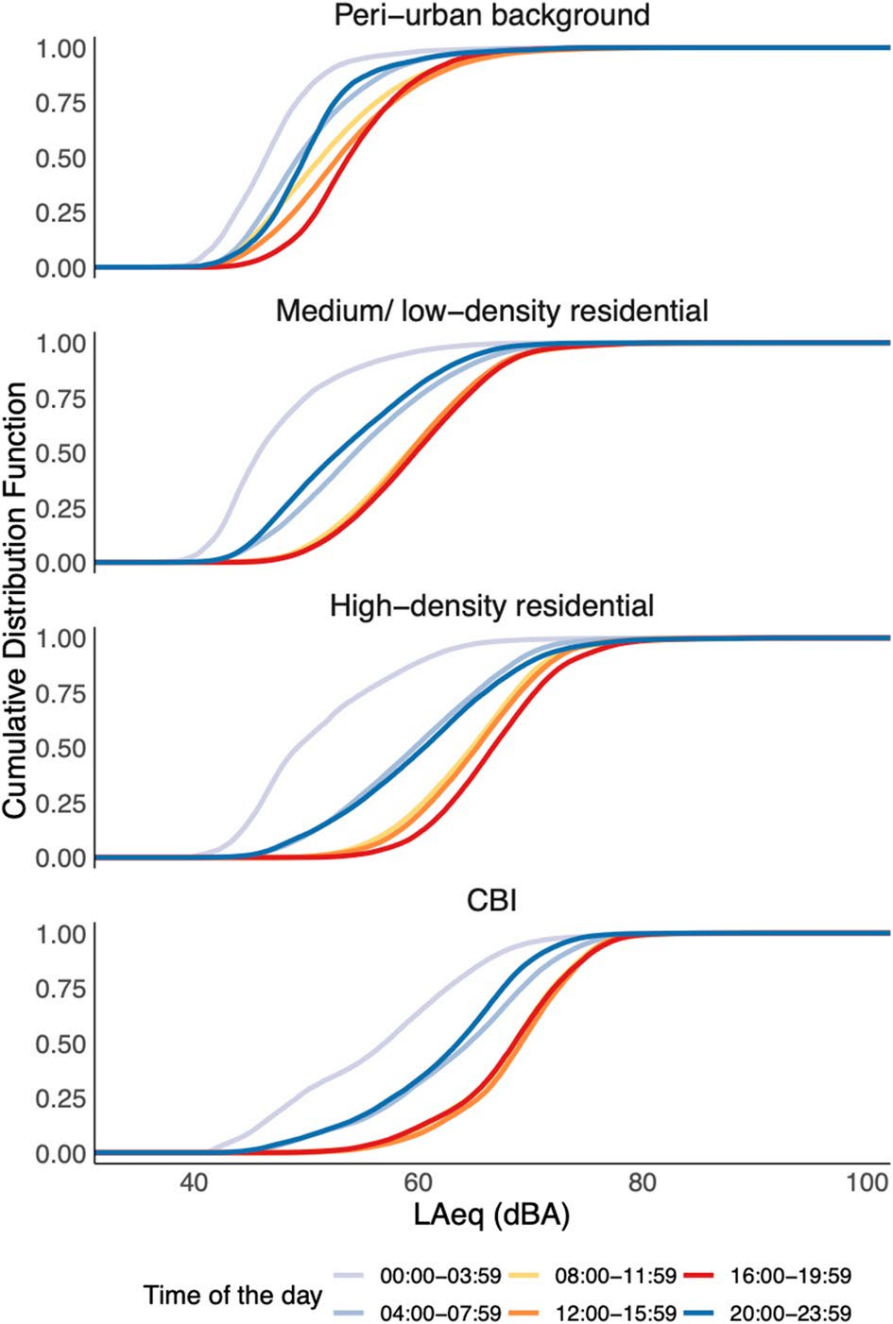
Cumulative density (distribution) functions (CDF) of 1-min sound levels (dBA) across a 24-h day period in the Greater Accra Metropolitan Area. Data from rotating sites. Blue lines represent time periods in the nighttime and early morning (8 pm–8 am) and red and orange lines represent periods in the daytime (8 am–8 pm). CBI: Commercial, business, industrial areas; LAeq: 1-min A-weighted equivalent continuous sound level; dBA: A-weighted decibels.

Sound levels were generally lowest between midnight and 4 am and highest between 8 am and 8 pm (S2), though the evening descent in sound levels started earlier at peri-urban sites (7 pm). There was also no singular daytime or nighttime hour which consistently had the highest or lowest sound levels. As well, the difference between daytime (L_day_) and nighttime (L_night_) was not consistent across land use types (Fig. 2). In general, the distribution of hourly sound levels (LAeq_1hr_) were similar across days of the week (Monday–Sunday) and between weekdays versus weekends for all sites (S2). Though there were some deviations, notably one of the high-density residential fixed sites had higher median day-time sound levels on Wednesdays (*p* < 0.01), which are known to be market days (S2). We also did not observe any substantial month–month sound level patterns when the yearlong fixed sites were stratified by land use area. This was also generally the case for each fixed site individually, with a few exceptions that were accounted for and described in greater detail in the Supplemental Information S2. Finally, during the annual one-month ban on ‘drumming and noise-making’ in the city as part of the traditional *Homowo* festival (May 13th–June 13th 2019), we found minimal changes in median hourly sound levels (LAeq_1hr_) at yearlong fixed sites along major traffic routes and in medium/low-density and peri-urban residential areas, with the exception of the two high-density residential sites, where the median levels during the ban were 2.7 dBA lower (*p* < 0.001) (S3).

### Spatial and temporal patterns of sound sources

**Figure 3:**
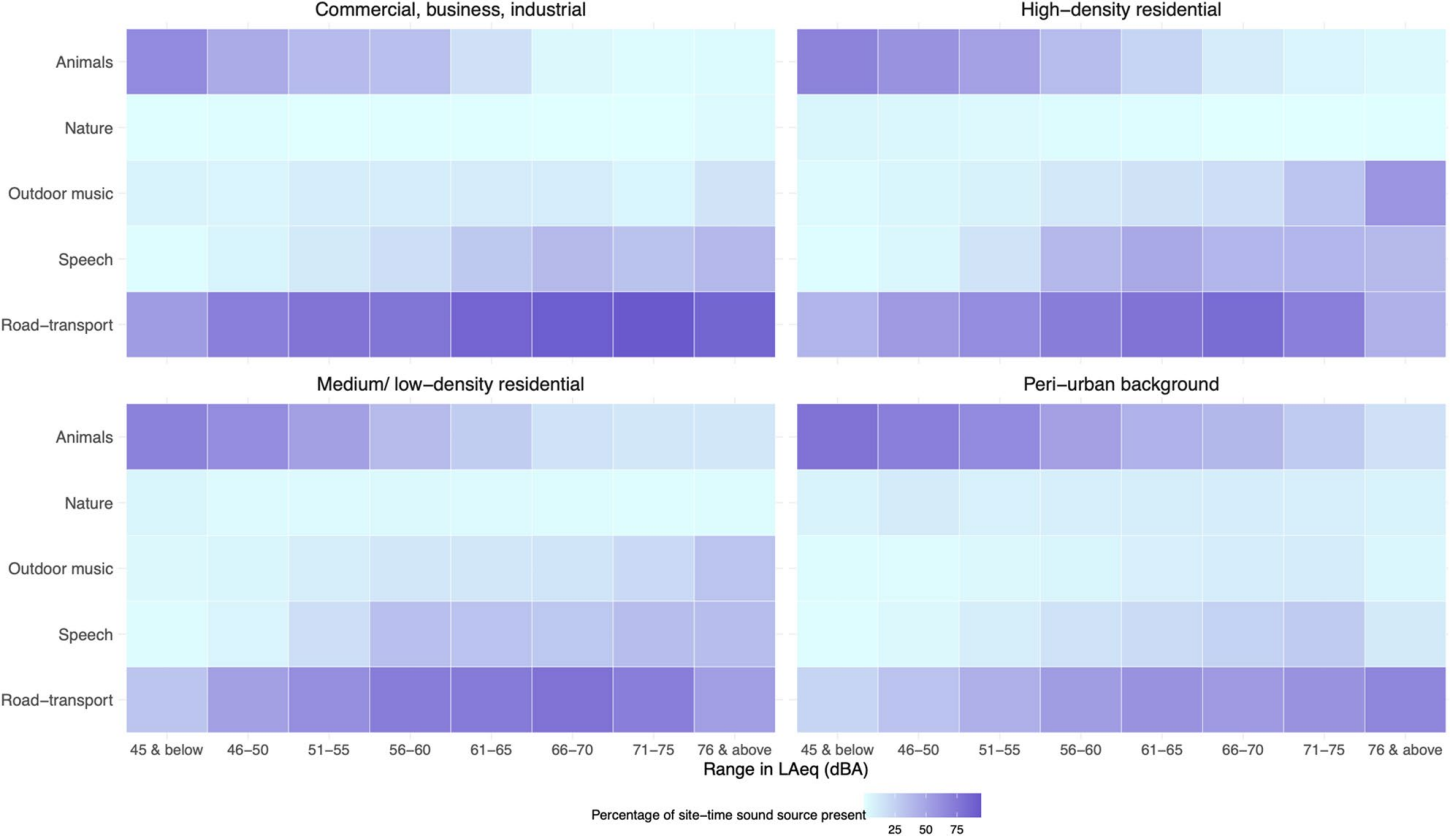
Prevalence of sound sources within land use areas and varying sound levels (LAeq_1min_) in the Greater Accra Metropolitan Area. Data from rotating sites. The percentage of site-time that each sound source was present was calculated from the data within each land use area and sound level interval separately.

As determined from the neural network, the most prevalent source of sound detected across sites in the GAMA was road-transport, which was present 64% of site-time among weeklong (i.e., rotating) sites (Table 2). Within CBI areas, road transport sound dominated (present 85% of site-time) and were the least prevalent within peri-urban areas (present 40% of site-time). Animal and insect sounds were also fairly common within medium/low-density residential and peri-urban areas (present 43% and 65% of site-time) but generally at lower sound levels (Fig. 3). Within high-density residential areas, human speech and outdoor music were fairly prevalent (present 33% and 15% of site-time) and generally at higher sound levels (Fig. 3). Nature sounds were uncommon across all land use areas (overall 4% of site-time present) and at all sound levels.

**Table 2:** Detection of sound source prevalence across land use areas in the Greater Accra Metropolitan Area*.

	Road-transport (%)	Animals and insects (%)	Outdoor music (%)	Human speech (%)	Nature (%)	Aircraft (within flightpath)**	Aircraft (outside flight path)**	Other (%)
**Rotating sites**								
Greater Accra Metropolitan Area	64	39	10	23	4	29	14	39
Accra Metropolitan Area	78	24	13	29	2	29	15	35
Land use***								
Peri-urban background	40	65	4	8	9	–	–	41
Medium/ low-density residential	64	43	10	22	3	–	–	40
High-density residential	71	30	15	33	2	–	–	40
Commercial, business, industrial	85	16	9	28	1	–	–	31
Fixed sites****	82	16	16	29	1	–	–	32

Data are expressed as the percentage (%) of site-time present.

*The top three sound classes based on modelled probability of presence were retained for each 10-s audio clip.

**Within/outside of 1 km of take-off/descent flight path for Kotoka airport (n = 7 rotating and n = 1 fixed site near flight path).

***Medium/low-density residential are typically formal residential areas; high-density residential are typically informal residential areas and could be classified as shantytowns or slums; peri-urban background are the least built-up areas and typically have an abundance of forest, grass land, shrubs, barren land, and/or water; commercial, business and industrial areas are places which are dominated by commercial, business, industrial, and/or government activities (see full criteria in S8).

*** 9 fixed sites had 3 CBI areas along major motorways, 2 sites in high-density residential neighborhoods, 3 sites in medium/low-density residential areas and one at a peri-urban location.

**Figure 4:**
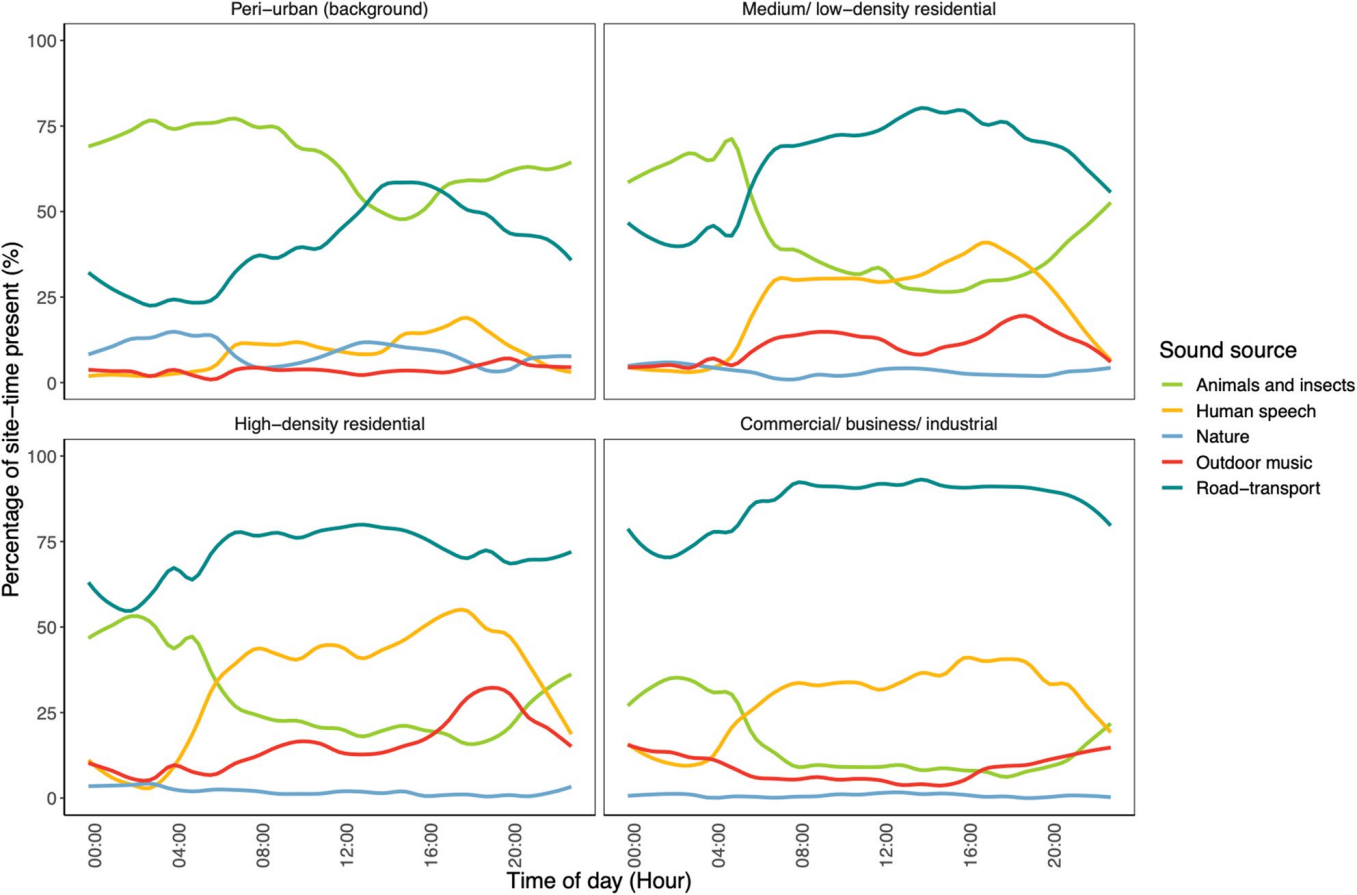
Diurnal patterns of the percentage of site-time that detected road-transport, animal and insect, human speech, outdoor music and nature sound sources were present for each hour of the day and within land use areas in the Greater Accra Metropolitan Area. Data are from rotating sites, but fixed sites had similar trends.

The prevalence of road-transport sounds within the GAMA was higher in the daytime (71% site-time present) compared with the nighttime (53% site-time present) (Fig. 4). This was also the case within CBI areas (90% (day) vs 77% (night) of site-time present), though the day and night-time differences were less pronounced (Fig. 4). As expected, road-transport sounds were prevalent along major traffic routes in the city in the daytime (80% site-time present) and night-time (72% site-time present). The day-time prevalence of road-transport sound was also inversely correlated with day-time Intermittency Ratios (Pearson correlation coefficient: − 0.50). The prevalence of animal and insect sounds was highest during nighttime, most notably in peri-urban areas. Human speech sounds and outdoor music increased in the morning and declined in the evening, most evidently within residential areas (Fig. 4). Outdoor music sounds were more prevalent on Sundays (12% site-time present) compared with other days of the week. We also found that human speech sounds were more prevalent between April and July compared with other months (S4).

### Short-term sound level measurements (generally) represent long-term levels

To evaluate whether short-term sound level measurements of community noise in Accra could represent longer-term ‘average’ levels, we assessed whether daily sound levels (LAeq_24hr_) from randomly sampled days were representative of yearly average levels for each fixed site separately. We found that daily measurements at each fixed site were generally representative of an entire year at those sites. Pooling results from all fixed sites together, we found that the median difference (median absolute difference) and the median percent difference (median absolute percent difference) were − 0.04 dBA (0.72 dBA) and − 0.06% (1.03%), respectively. However, the magnitude of the deviations did vary between fixed sites and there were some outlier days. For example, our three CBI fixed sites that were situated along major roads/highways had median differences of 0.19 dBA, − 0.16 dBA, and − 0.63 dBA, respectively. The sites with greater deviations between short-term measurements and long-term averages were the high-density residential sites (median differences of 2.59 dBA and − 0.58 dBA). A table with results shown for each fixed site is in the supplementary information (S5).

## Discussion

SSA cities are growing rapidly amid a rising challenge of environmental pollution, including noise pollution. Like many other SSA cities, community noise in Accra is increasingly becoming a public health concern ^34–36^, but there is scare evidence on the levels, patterns, and sources to support noise management strategies and targeted interventions. Using rich data from a yearlong field measurement campaign, we descriptively characterized the spatiotemporal patterns of the sound environment in the Greater Accra Metropolitan Area. Day and night-time sound levels in most parts of the GAMA surpassed national and international health-based guidelines ^37, 38^. Commercial, business, industrial areas had the highest median daily sound levels as well as the highest prevalence of road-transport sounds. Peri-urban areas had the lowest measured sound levels and the highest prevalence of animal sounds, particularly during the night-time. Human speech and music sounds were detected most often at higher sounds levels and in residential areas, particularly in high-density areas. In Accra, stronger regulation and policy on urban noise, which considers the total acoustic environment, should be implemented.

We found that sound levels and trends across land use in Accra reflected what was found in a previous study in South Africa ^6^. We also found that the majority of measured day and night-time sound levels in the GAMA exceeded the Ghana Standards Authority limits for community noise in comparable areas (Supplementary Information S6) ^37^. At the regional level, there are no environmental noise guidelines set for Africa by the World Health Organization (WHO) to compare our results. When compared to the WHO European guidelines, 98% of night-time sound measurements (L_night_) in the GAMA and 99% in AMA were higher than the guidelines for long-term exposure to L_night_ from road-transportation sources (guideline: < 45 dBA) ^38^. Evidence primarily from Europe has found that night-time sound above 45 dBA from road-transport is associated with adverse health impacts, particularly to sleep disturbance ^38^. Though, we recognize that this comparison is made to a source-specific guideline (i.e., road-traffic noise) and our source analysis from the audio recordings showed that, while we measured along the roadside, we captured community noise comprised of sound from multiple sources. We also recognize that this comparison is made to a guideline set for a different region of the world, which may have cultural and contextual differences affecting noise sources and levels. Not to mention, overall noise levels in SSA cities ^6, 14, 15^, such as Accra, may be higher than in European cities ^5, 16, 20, 39^.

Health impacts of noise, such as annoyance and sleep disturbance, depend not only on levels but also on other characteristics, such as eventfulness and intermittency. For example, intermittent environmental noise has been found to have a larger effect on sleep disturbance than continuous noise at the same level ^40, 41^. Recent evidence from Switzerland also suggests that for night-time road-traffic noise above 60 dBA, higher Intermittency Ratios modify the exposure response-curve of the probability to be highly sleep disturbed in a positive direction ^42^. Across our measurement sites in the GAMA, IR_night_ levels varied widely (IQR: 35%, 67%), though the median level was lower than what was previously reported among a Swiss cohort of adults exposed to road-traffic noise (median IR_night_: 73%) ^43^, likely as a result of the higher overall noise levels observed in Accra. Though along major traffic routes, low-levels of day-time IR were similar to a study in Milan (Spain) ^44^. We can also only interpret our results within the context of the fixed event cutoff that was used. If we had interpreted our main results with stricter cutoffs, the intermittency of environmental noise in the GAMA would have appeared lower. Future epidemiological work in SSA cities, such as Accra, is needed in order to estimate the exposure–response relationships between noise levels, health outcomes, and the potential modifying effects of noise intermittency. Particularly as the sources and characteristics of noise in SSA cities may be unique and the majority of the evidence is from North America and Europe.

We found that daily sound level measurements at the majority of the fixed sites were generally representative of an entire year, as has been found elsewhere ^45–47^, though some outlier days did show deviation from the yearlong average. This finding is useful for future studies, particularly in SSA, as the sampling duration could be reduced from what we conducted in Accra. Sites with the most variability, such as the high-density residential sites, had some of the greatest differences in daily sound levels between short-term measurements and yearlong averages. While sites with the least variability, such as those along major traffic routes, were largely consistent. In a city like Accra, multiple sequential or random days of measurements at each site would ideally ‘average out’ sound levels captured on outlier days. Previous studies have even shown that repeated short-term measurements conducted for several minutes/hours can represent long-term trends ^46^. Future work is needed in SSA cities to verify if that finding applies in this setting, given that there may be contextual differences impacting sound sources and their temporal variations.

While road-transport sounds were the most prevalent in the GAMA throughout the day, reduction of noise pollution from road transport sources is not prioritized by local policy actions. Recent measures to curb road-transport related air pollution in Ghana, such as the regulation and taxes imposed on the import of old (and often noisy) vehicles into the country, will likely have an indirect impact on road-transport related noise ^48^. Policies that specifically target noise sources, such as banning or restricting excessive vehicle honking or restricting access of heavy vehicles to densely populated areas, could also help reduce road-transport noise in the GAMA ^49, 50^. Implementing direct changes to the urban environment such as modifying pavement material ^49, 51^ could curb some road-transport noise, particularly on higher-speed roads ^52^. In AMA, 20% of roads are still unpaved, particularly in the poorer neighborhoods ^53^. If and when new highways/motorways are being designed and built, distance or noise barriers can be built between road networks and buildings ^49^. However, street level noise barriers need to be considered carefully in a SSA context in terms of its potential adverse economic impact on street hawkers and informal vendors who rely on access to commuters for road-side commercial activities ^54^. Finally, the GAMA has over a fifth of Ghana’s population but 42% of its private vehicles ^55^. Thus road-traffic noise could be reduced in the GAMA with changes to urban planning that inspire modal shifts towards cycling and walking and delivery of services that encourage mass transit. Future work is needed to evaluate the listed noise abatement initiatives in Accra and within a SSA urban context at large as almost all of the evidence is from high-income countries ^56^. Future studies in Accra and other African cities ^57^ could treat changes to road-networks, transportation services, and/or transport policies as natural experiments to investigate the potential noise level and health impacts in these settings. For example, if Accra’s failed Bus Rapid Transport system is eventually executed ^55^, a study could be conducted to investigate its potential impact on road-traffic noise (i.e., by boosting the use of mass transit and thereby reduce the number of private vehicles) on the roads.

Another source of transportation-related noise comes from airplanes ^42, 58^, which has been linked to numerous adverse health outcomes, primarily in Europe ^7, 42, 59^. Although the neural network over predicted airplane sounds in Accra with a high degree of error, it still seemed to be able to differentiate the impact of this source between areas near and far from the airport. Aircraft traffic near Kotoka International Airport (KIA) may already be a source of noise pollution for the people who live or work near the airport ^18^ and is likely to only get worse as the demand for air travel increases due to Ghana’s open skies policy and expansion of KIA. Both initiatives have resulted in increases in aircraft movement (international) from 17,481 in 2008 to 26,726 in 2017 ^60^.

While the focus in North America and Europe is primarily on transport-related sources of noise, we found that outdoor music and human speech sounds were quite common in residential areas in Accra. Loud music from a variety of sources and activities, such as small scale business and shops, festivals and events, religious activities, bars, nightclubs and restaurants are commonplace in Accra and other SSA cities ^3, 14, 61, 62^. Previous studies found that perceptions of music and speech sounds varied widely between countries ^25, 63^. In Accra, religious activities in particular can generate high-levels of outdoor human speech and music sounds and has received local and international press attention ^3, 34, 64, 65^. Religious places of worship dot SSA cities in the thousands and are a common source of community sound ^3, 64–66^. Enforcement of sound generated from religious activities may be difficult in SSA, as environmental protection agencies in cities need to monitor thousands of places of worship which can range from small informal gatherings to over 200,000 person mega congregations ^64^. Furthermore, policy makers need to carefully balance considerations around religious freedom of expression and infringement of public health ^3^. Some SSA cities have tried to enforce removal of external speakers from religious buildings and enforce building codes for soundproofing ^64^.

Nature (e.g., running water) and some animal sounds (e.g., birdsong) are generally considered as a ‘tranquil’ soundscape and have been linked to enhanced feelings of wellbeing and markers of stress reduction in experimental studies ^27, 67–69^. These sounds were also identified as ‘pleasant’ among adult participants in a small-scale study conducted in Cape Coast, Ghana ^70^. From our Accra audio recordings, nature and animal sounds were classified most often in quieter peri-urban and medium/low-density residential areas. It is possible that the inverse relationship between animal sound presence and measured sound levels is due to animals escaping locations with noise and human activity ^71^ as well as features of the peri-urban environment which is less built up than other areas of the city. Though, it is also possible that part of this observed relationship is due to the masking effects ^72^ of other ‘louder’ sounds picked up by the neural network. Urban planners in Accra can consider ways to protect and increase public parkland and green spaces in the GAMA which could generate and facilitate acoustic environments that are abundant in natural and animal sounds. Though, there is clearly a need for research to be done on soundscapes in Accra and other SSA cities. Spatial and temporal information on sound source prevalence, sound levels and population perceptions of different sound environments can help guide strategic policy making and urban planning to protect those soundscapes which are most tranquil/pleasant or reduce sound source emissions which are perceived locally as the most harmful/ annoying ^73^. There is also potential for citizen science approaches to support future soundscape research in this setting, by using mobile phone applications to gather information on sound perceptions and characteristics ^73, 74^.

Notable strengths of our study include the analysis of data from a comprehensive and large-scale community sound measurement campaign in a SSA city, which included weeklong and yearlong measurements at 136 and 10 representative sites in Accra. Our estimates of the Intermittency Ratio in addition to the more commonly reported metrics of equivalent continuous sound provide a broader picture of the characteristics of community sound in Accra. Our use of audio and a deep neural network provide information on the spatiotemporal patterns of a variety of sound sources in an urban SSA context, which further illustrates the potential for this approach to assist with urban noise planning and management. Given the high spatial and temporal resolution of our data, a future area of research could be to spatially model and interpolate sound levels, and even source presence, across areas of Accra through geostatistical modelling methods.

The data used in this study also have a number of limitations that are common to many field research studies. Our data collection campaign was interrupted due to the COVID-19 pandemic lockdown in Accra. Consequently, we collected additional data a month beyond our planned campaign after the lockdown was lifted. Analysis of fixed sites showed that post-lockdown sound levels in the city returned to pre-lockdown levels (more information in S7). The land use classifications by which we stratified our results were adapted from a city-wide dataset ^75^ combined with field observation of each measurement sites (S8); this approach was subjective and could result in a lack of reproducibility for future studies. Furthermore, as the greatest variability in day and night-time noise was concentrated in high-density residential land use areas, future sampling strategies which over-sample in these types of areas, might be warranted in an urban SSA context. For storage and privacy reasons, we collected 10-s audio clips every 10-min (i.e., 6 times in an hour, every hour of the day, every day of the week). We thus assume that our temporal sampling strategy provides an adequate representation of the underlying distribution of sound source frequency over time. Furthermore, our approach to identifying a variety of sound sources through the collection of urban audio recordings may not be feasible in all research settings with different privacy frameworks and laws. Alternative methods that have been proposed are based on fast response L125ms spectral data. Though these would require use of more sophisticated and expensive equipment and may restrict the quantity of data available for analysis. Our accuracy metrics for classifying sound sources in the audio recordings might improve if we had a) developed and trained our own model or b) trained the model on similar data from the same geographic context as our own audio recordings. Finally, we can only speculate with evidence from studies conducted outside of SSA on how different sound sources are perceived as pleasant/tranquil, unwanted (noise), or neutral by residents of Accra as we did not collect information on people’s preferences and perceptions alongside the audio recordings.

## Conclusions

Like many other SSA cities, Accra is growing rapidly amid a growing challenge of environmental pollution, including rising community noise levels. From a large-scale and comprehensive community noise measurement campaign, we characterized sound levels, patterns, and sources across the Greater Accra Metropolitan Area. The data collection and characterization approach may be applied to other cities in SSA and globally to support noise management strategies, targeted interventions, and to provide additional characterization for application to epidemiologic studies. Our results showed that sound levels in Accra largely surpassed local and international health-based guidelines. Road transport-related sources were the main contributor to community sound, though mixtures of sources were present. Stronger regulation and enforcement are needed in Accra in order to ensure that permissible limits, which are in place to safeguard public health, are met. Policy makers can also partner with traditional authorities in Accra, who play an important role in civil government, in managing community noise.

## Methods

### Study design

A detailed description of the measurement campaign protocol, which was designed for both noise and air pollution, is found elsewhere ^76^. In brief, we designed a 12-month campaign, which started in April 2019, to capture both the spatial patterns and temporal trends by monitoring at ‘fixed’ (1 year, n = 10 sites) and ‘rotating’ (7-day continuous, n = 140) sites. The field campaign was interrupted in its last scheduled month (April 2020) due to the COVID-19 pandemic and the subsequent lockdown of Accra. After the lockdown was lifted with less restrictions on movement and daily life returned to normal, we collected measurements at 12 additional rotating sites (between May and June 2020), thus covering a total of 136 of the 140 planned rotating sites.

The ten fixed monitoring stations were purposefully chosen to represent areas with a diversity of geographical features, including land cover ^75^, population density ^77^, road-networks ^78^, and neighborhood socio-economic status and biomass fuel use ^77^. The rotating sites were selected by a stratified random sampling approach where potential locations were randomly distributed within the GAMA across strata of land cover ^75^, with more emphasis placed on AMA, which is the most populated area. A detailed description of the protocol for sampling measurement sites and rational can be found in Clark et al., 2020 ^76^. There are other approaches for selecting measurement sites, such as stratifying by road-network type, especially where traffic is the dominant source of noise ^79^.

### Sound level measurement and audio

We used the Noise Sentry sound level meter datalogger (NSRT_mk3) from Convergence Instruments, Canada (https://convergenceinstruments.com) to measure A-weighted sound levels (decibels (dBA)) ^76^. The digital MEMS microphone reduces the cost of the sound level meter but complies with Type 1 precision standards ^80^. The sound level meters were programmed to continuously capture sound levels which were integrated and logged every minute. Prior to each monitoring session, the sound level meters (SLM) were calibrated with a CA114 sound calibrator at 94.0 dB ± 0.3 dB and 1000 Hz ± 0.5% (Convergence Instruments, Canada). The Noise Sentry’s do have a noise floor of 30 dBA, though this is not a limitation of our study, which aimed to characterize the average noise trends in Accra across a large-scale. As well, pilot measurements conducted at 9 diverse locations in the city showed that even the ‘quietest’ site did not have an LAeq_1min_ value which went below 34 dBA. Our SLMs were also field validated in a separate aircraft noise study conducted in San Francisco against a sophisticated Type I instrument (DUO 01 dB) ^81^ and the agreement was high (mean and median second by second difference between the instruments was − 0.42 and − 0.38 dBA, respectively). In measurements conducted prior to our study, we also found good agreement between our SLMs and a Cirrus Optimus Red where the difference between monitoring period LAeq’s were less than 1 dBA. We also deployed low-cost audio recorders (AudioMoth, Open Acoustic Devices (Oxford, UK)) to record samples of the outdoor acoustical environment for analysis of sound sources. The audio recorders were co-located and recorded for 10 s every 10 min. We collected a temporally representative sample of the 10-s-long audio clips in order to represent the underlying distribution of sound source frequency over time. Specifically, we collected audio throughout the day (6 samples per hour, every hour) and across days (Monday–Sunday) at each measurement site in the city. For the fixed sites, we additionally have data spanning 12 months of the year. Collecting audio for 10-s snapshots also preserves individual privacy.

At each measurement site, the monitors were housed in a portable, weather and rainproof enclosure that was custom built to house and protect the study instruments (see study protocol for additional information ^76^). Depending on the specific site, the field staff either attached the enclosure to lightweight poles that were mounted on flat rooftops/balconies on one-story buildings or mounted it on an existing pole secured directly in the ground (e.g., lamppost). Equipment was installed at ~ 4 (± 1) meters above ground, with no obstructions between sound monitors and sources, and the nearest façade was at least 2 m away. Equipment was programmed to run continuously for 7-day periods, after which they were either swapped out and installed at a new set of rotating sites or replaced with a fresh batch at existing fixed sites. Although this analysis did not consider weather conditions, weather monitors were installed at 6 fixed sites and the data will be incorporated into future modelling studies. Finally, we did not install an audio recorder at one fixed site near the ocean as initial monitors were damaged from the contact of the airborne sea-salt with the exposed circuit board.

At each site, trained field staff documented information about the set-up, such as the start and end time of monitoring and the height of the equipment off the ground. Based on a similar classification as the land cover data use to select site locations ^75^, our research team reclassified each site as to its current land use state by consensus and following a set criterion (S8). The four classes included medium/low-density residential; high-density residential; peri-urban ‘background’ (e.g., forest, agricultural land); and commercial, business, and industrial (CBI) areas. Briefly, medium/low-density residential are typically formal residential areas and can have paved or unpaved roads which are double or single lane. The housing property can have yards, fences, walls, and/or driveways and there is a clear demarcation of where one home ends and another begins. High-density residential are typically informal residential areas and could be classified as shantytowns or slums. Roads (paved or unpaved) are typically narrow, sometimes unidentifiable, and buildings are typically small with a lack of a clear demarcation of where one home ends and another begins. Peri-urban ‘background’ are the least built-up areas and can have an abundance of forest, grass land, shrubs, barren land, water, and/or be classified as a park. CBI areas are places which are dominated by commercial, business, industrial, and/or government activities. These areas can have large buildings, though that is not a requirement (see full criteria in S8). The following number of sites fell within each land use category: CBI (rotating: 25, fixed: 3), high-density residential (rotating: 30, fixed: 2), medium/low-density residential (rotating: 52, fixed: 4), peri-urban (rotating: 29, fixed: 1). Seven of the sites were along a major road (e.g., highway/ motorway), 34 sites were along a secondary/tertiary road, and the rest were along minor roads. To provide additional contextual information on our land use areas, and in particular how built up and populated they are, we overlayed the locations of our measurement sites onto city-wide geographic datasets of population density and vegetation in the GAMA (Table 3). Specifically, population density (people/km^2^) within census enumeration areas (median EA area: 0.03 km^2^) was obtained from the most recent Ghana National Population Housing census (2010) ^77^. This is the most up to date and reliable record of population distribution within Accra at the small-area level ^77^. We obtained estimates of vegetation by calculating the Normalized Difference Vegetation Index (NDVI) from 30 m spatial resolution Landsat 8 satellite imagery collected at the mid-point of the campaign in January 2020 ^82^. NDVI values close to zero indicate no green vegetation (mostly built-up areas), and values close to + 1 indicate a high abundance of available green vegetation.

**Table 3:** Population density and vegetation at measurement sites.

	High-density residential	Medium/low-density residential	Commercial, business, industrial	Peri-urban background
Normalized Difference Vegetation Index (NDVI) (value range: 0–1)*	0.09 (0.03)	0.13 (0.04)	0.10 (0.03)	0.22 (0.06)
Population density (people/km^2^)	35,833 (42,011)	6881 (9612)	7966 (9220)	1701 (3867)

Data are summarized as means and standard deviations within land use areas.

*To capture vegetation in the area surrounding each site, we created a 200 m radius buffer around the site and calculated the average NDVI within it. NDVI can be a negative value, which would indicate water, though at all of our measurement sites, NDVI levels were positive between 0 and + 1.

After cleaning the data, we had 952-site days of sound level measurements from rotating sites (n = 136) and 3320 site-days from fixed sites (n = 10). Four of the fixed sites each lost a week of data due to equipment malfunction. We use 1,232,640 site-seconds and 1,370,880 site-seconds of audio data from rotating (n = 121 sites) and fixed sites (n = 9). Missing data was due to equipment malfunction, damage, or theft. Pre-campaign sound level monitor-monitor precision tests showed good agreement (see our protocol paper for more details ^76^) and the duplicate measurements that we conducted throughout the campaign at 16% of rotating sites indicated that the monitors did not drift from each other over time (see S9 for more information).

### Data analysis

#### Sound level and event metrics

We calculated the following sound level and intermittency metrics for each site and date of measurement:

- A-weighted equivalent continuous hourly sound levels (LAeq_1hr_ (dBA));
- A-weighted equivalent continuous daily sound levels (LAeq_24hr_ (dBA));
- A-weighted equivalent continuous daytime sound levels between 6:00 and 21:59 h (L_day_ (dBA));
- A-weighted equivalent continuous nighttime sound levels between 22:00 and 5:59 h (L_night_ (dBA));
- Daily intermittency ratio (IR_24hr_ (%))
- Daytime intermittency ratio between 6:00 and 21:59 h (IR_day_ (%))
- Nighttime intermittency ratio 22:00–5:59 h (IR_night_ (%))

We calculated sound metrics for each site and date of measurement so that variability within-and between sites over time could be characterized. The equivalent continuous sound level represents the steady sound level that has the same total energy as the fluctuating sound over a given period of time. In contrast, the Intermittency Ratio (IR) expresses the amount of intermittent sound in a defined period of time. Essentially, the IR discriminates between sound environments with marked peaks and without. Intermittent environmental noise in particular has been found to have a larger effect on sleep disturbance than continuous noise, particularly from noise events that emerge from the background level ^40, 41^. The IR is defined as the percentage of sound energy in the total energetic dose that is created from distinct sound events that exceed a certain threshold ^83^. By definition, the IR is bounded between 0 and 100%. To define a sound ‘event’, equivalent continuous sound levels recorded for each minute of measurement (LAeq_1min_) had to surpass a fixed cut-off of three dBA above the site and date specific daily, daytime or nighttime equivalent continuous sound level. Three dBA was chosen as it has been shown to produce IRs that can distinguish between situations with different degrees of intermittency ^83^. The equation used to calculate the IR is in the supplementary information (S10). As a secondary analysis, we also computed IRs using alternative fixed cut-off values of + 4 and + 5 dBA.

#### Sound source classification

Research on environmental sound type recognition, which includes human, natural, and mechanical sounds, have increased in the past decade ^84^, and the use of Machine Learning techniques has been a major part of that progress ^85, 86^. Within our study, to classify the different types of sound sources present in the GAMA, we applied a pre-trained neural network model (DEEP-Hybrid DataCloud project ^87^ based on Changsong et al. 2018 ^88^) to our outdoor audio recordings. This is an open source model that was pre-trained on the *Audio Set* dataset published by Google ^89^. *Audio Set* consists of 10-s manually labelled YouTube videos and a structured hierarchical ontology of 527 sound classes. *Audio Set* is *weakly labelled* meaning that only the presence or absence of audio events are known in a clip. The neural network is a Multi-level Attention Model as described by Yu et al. ^88^, which takes a spectrogram of each audio clip as an input to several, sequential fully connected layers, whose final and intermediate outputs provide inputs in turn to several “attention” modules, designed to selectively ignore irrelevant sound segments such as background noise while attending to those segments of the sound most relevant to classification after training. The final classification is derived from the concatenation of the outputs from the attention modules.

We applied our own audio recordings to the pre-trained model. As is expected in an urban sound environment, the presence of sound classes in each audio recording was not mutually exclusive as multiple sound classes were detected within each audio recording. For each 10-s recording, we obtained the model’s top three predicted sound classes; thus, we call these ‘detected’ sound classes. For interpretability, we then grouped the sound classes into higher order sound source categories that were adapted from ISO standards for acoustic environment characterization ^90^: road-transport sounds (e.g., motorized vehicles), animal and insect sounds (e.g., wildlife or domesticated animals), outdoor music, human speech, airplane sounds, nature sounds (e.g., wind, rain, thunder), and ‘other’ sounds (more details on the neural network model in Supporting Information S11). The accuracy of the neural network model’s predictions in each sound source category was tested by comparing agreement against manual sound labels applied by a researcher that was blinded to the model results from 150 randomly selected audio files. The positive predictive value (number of true positives/(number of true positives + false positives)) was moderate-high for outdoor music (71%), road-transport (76%) sounds, human speech (83%), animal and insect sounds (97%), and nature sounds (100%), however the model performed poorly for airplane sounds (4%) (more information in S11). Given the structural and contextual differences between the data the neural network was trained on (e.g., YouTube videos) and our own data (street-side recordings of the total sound environment), we found the accuracy metrics to be acceptable for the purposes of our study. We also investigated whether airplane predictions were more common near the airport by separating sites that were within 1-km around Accra’s Kotoka International airport (KIA) flight path and sites that were outside. The flight path for this analysis represents the landing/ take off trajectory of low-flying planes coming into/ going away from the airport.

#### Descriptive summaries

We report summary statistics on the spatial and temporal trends in sound level, sound intermittency, and sound source prevalence from the fixed and rotating sites separately. We assessed spatial trends by mapping the data to each site’s coordinate (Latitude/Longitude) location and by stratifying the data on land use characteristics as well as near major roads ^78^. Temporal trends were evaluated at multiple time periods, including minute by minute, hourly, daily, weekly, and monthly aggregates. The sound level and intermittency metrics were summarized as medians and interquartile ranges (IQR) in aggregate and within spatial and temporal strata. The probability that strata were different were assessed with non-parametric difference in medians tests, specifically the Wilcoxon Rank Sum. We also described time trends for the 10-fixed sites individually.

We summarized the prevalence of sound sources across multiple time intervals within the GAMA, in AMA, within land use areas, near major roads, and for each individual measurement site. The percentage of time a particular sound source was present (i.e., prevalence) at any given site was calculated as the ratio between the number of audio recordings that had the sound source predicted as present (yes = 1, no = 0) over the total number of audio recordings collected during the monitoring period at that site, multiplied by 100. For summaries where sites were pooled into strata (e.g., land use) we considered the prevalence to be a measure of site-time as audio files captured across different time periods and locations (sites) were included in the overall metric. In order to characterize the co-occurrence of sound levels and sound sources in the GAMA, we linked the sound source and sound level data by site, date and time (hour and minute) of measurement. Specifically, we linked the sound level data, which was continuously recording and integrated sound levels every minute, to the audio recordings, which were collected for a period of 10-s once every 10-min. For this, we had to make the assumption that the 10-s audio sample was representative of the acoustic environment over that 1-min time period. We then categorized the sound level data into fixed intervals (e.g., LAeq_1min_: 51–55 dBA, 56–60 dBA), and for each sound source and land use area separately, calculated the percentage of site-time that the sound source was present within intervals of LAeq_1min_ (the distribution of sound levels for this analysis and the position of cut-points for intervals is in S12).

Finally, we assessed whether random days of measurement were representative of average yearly levels at fixed sites. This information is useful for informing the validity of noise pollution studies, particularly in SSA, that aim to characterize long-term levels with short-term measurements. We first selected 50 random days of LAeq_24hr_ measurements, 5 from each of the 10 fixed sites. We then calculated the dBA difference and percent difference (%) between the randomly sampled LAeq_24hr_ (n = 5 for each site) and the average yearly LAeq_24hr_. We conducted this comparison for each fixed site separately.

The audio classifier model was run in Python (Python version 3.6) while all other data analyses and data visualizations were performed in R (R version 3.6.3).

### Ethics

The protocol for the study was deemed exempt from full ethics review at Imperial College London and the University of Massachusetts Amherst; it was approved by the University of Ghana Ethics Committee.

## Data availavility

The processed data will be made available upon reasonable request to the corresponding author.

## Supplementary Information

Supplementary Information.
