# Supplementary material for: Space-time characterization of community noise and sound sources in Accra, Ghana
Source: Sci Rep. 2021 May 27;11:11113. doi: 10.1038/s41598-021-90454-6 (PMC8160008; doi:10.1038/s41598-021-90454-6)
Supplement: Supplementary file 1 — Supplementary Information. [file 41598_2021_90454_MOESM1_ESM.docx]

**Space-time characterization of community noise and sound sources in Accra, Ghana**

Supporting Information

Sierra N. Clark ^a,b^, Abosede S. Alli ^c^, Ricky Nathvani ^a,b^, Allison Hughes ^d^, Majid Ezzati ^a,b,e,f^,

Michael Brauer ^g^, Mireille B. Toledano ^a,b,h^, Jill Baumgartner ^i,j^, James E. Bennett ^a,b^, James Nimo ^d^, Josephine Bedford Moses ^d^, Solomon Baah ^d^, Samuel Agyei-Mensah ^k^, George Owusu ^l^,

Briony Croft ^m^, Raphael E. Arku ^c*^

^a^ Department of Epidemiology and Biostatistics, School of Public Health, Imperial College London, London, UK

^b^ MRC Centre for Environment and Health, School of Public Health, Imperial College London, London, UK

^c^ Department of Environmental Health Sciences, School of Public Health and Health Sciences, University of Massachusetts, Amherst, USA

^d^ Department of Physics, University of Ghana, Accra, Ghana

^e^ Regional Institute for Population Studies, University of Ghana, Accra, Ghana

^f^ Abdul Latif Jameel Institute for Disease and Emergency Analytics, Imperial College London, London, UK

^g^ School of Population and Public Health, The University of British Columbia, Vancouver, Canada

^h^ Mohn Centre for Children’s Health and Wellbeing, School of Public Health, Imperial College London, London, UK

^i^ Institute for Health and Social Policy, McGill University, Montreal, Canada

^j^ Department of Epidemiology, Biostatistics, and Occupational Health, McGill University, Montreal, Canada

^k^ Department of Geography and Resource Development, University of Ghana, Accra, Ghana

^l^ Institute of Statistical, Social & Economic Research, University of Ghana, Accra, Ghana

^m^ SLR Consulting, Vancouver, Canada

**S1. Correlation of day and night Intermittency Ratios (IR) and comparison of IR calculated with different event-thresholds.**

**
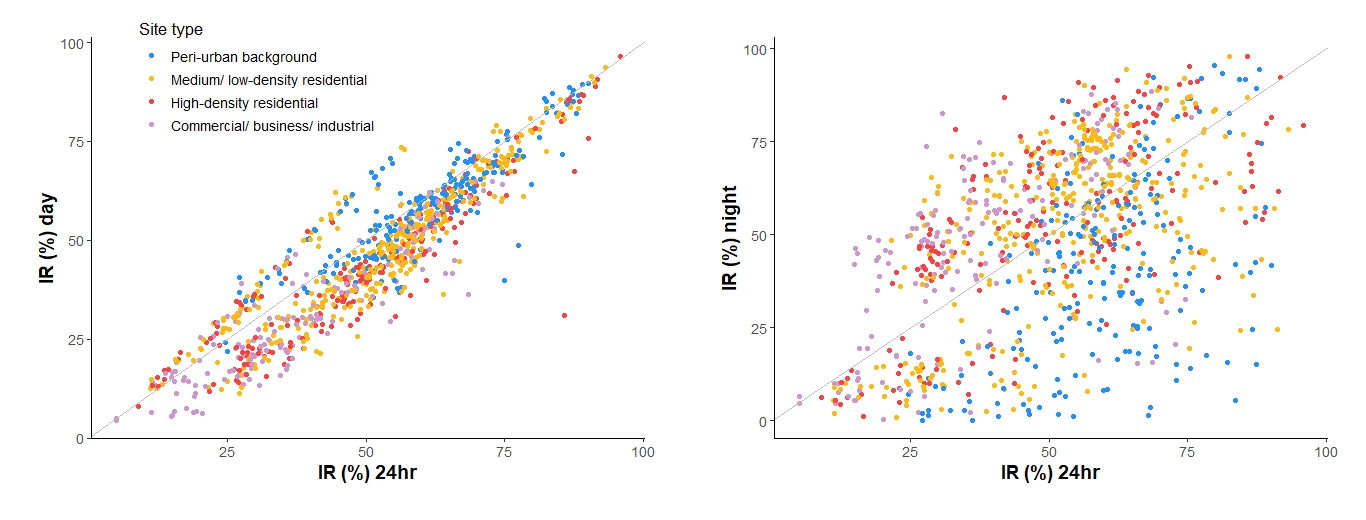
**

**Figure 1. Scatterplots of the relationship between IR_day_ with IR_24hr_ (left) and IR_night_ (right) with IR_24hr_ for each site and date of monitoring among the rotating sites in the Greater Accra Metropolitan Area.**

Correlation of IR_24hr_ with IR_day_ and IR_24hr_ with IR_night_ shows that IR_24hr_ is largely driven by IR_day_.

To define a sound event for the IR metric, equivalent continuous sound levels recorded for each minute of measurement (LAeq_1min_) had to surpass a fixed cut-off of +3 dBA above the site and date specific daily, daytime or nighttime equivalent continuous sound level. Three dBA was chosen as it has been shown to produce IRs that can distinguish between situations with different degrees of intermittency ^1^. As a secondary analysis, we also computed IRs using alternative fixed thresholds of +4 dBA and +5 dBA.

**Table 1. Pearson correlations between IR_24hr_ calculated with different fixed event thresholds.** Data from rotating sites.

|  | IR_24hr (+3dBA fixed threshold)_ |
| --- | --- |
| IR_24hr (+4dBA fixed threshold)_ | 0.99 |
| IR_24hr (+5dBA fixed threshold)_ | 0.96 |


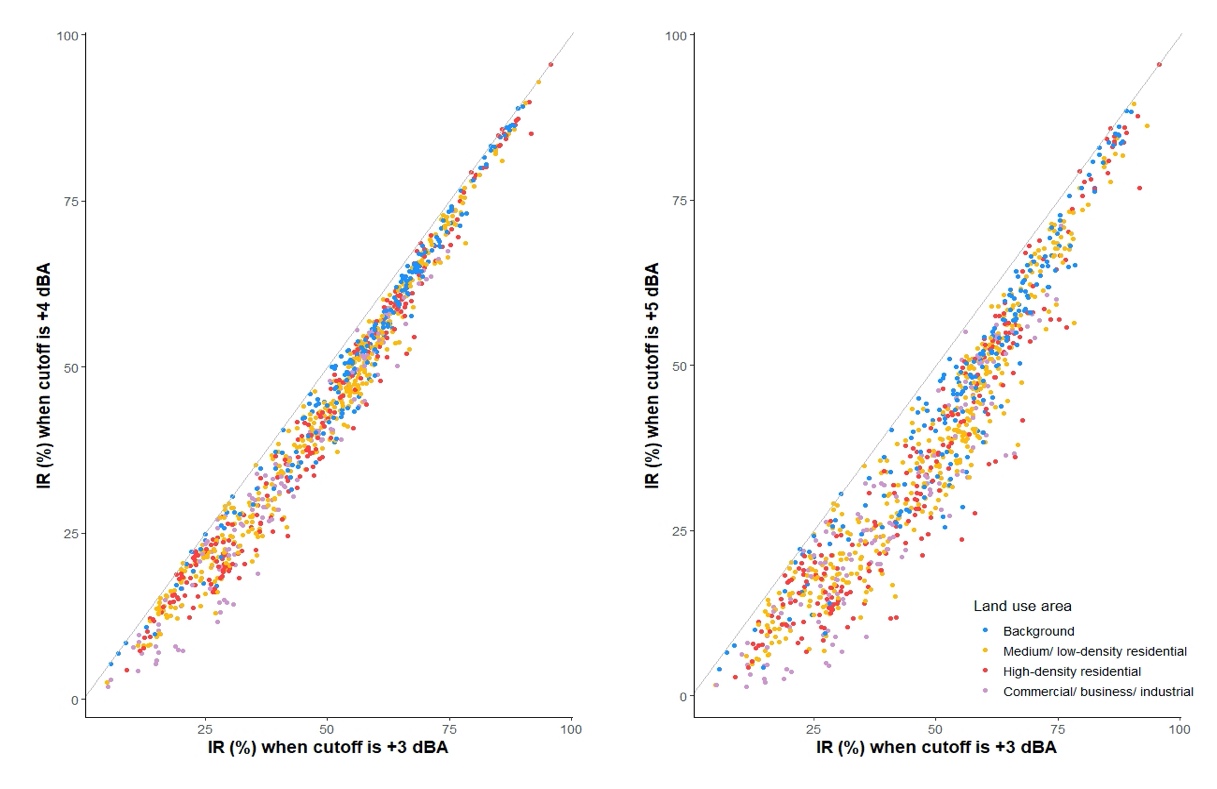


**Figure 2. Scatterplots of the correlation between IR_24hr_ calculated with different fixed event thresholds/ cutoffs.** Data from rotating sites. **Background** refers to Peri-urban background.

**S2. Temporal trends of sound levels and sources**

**
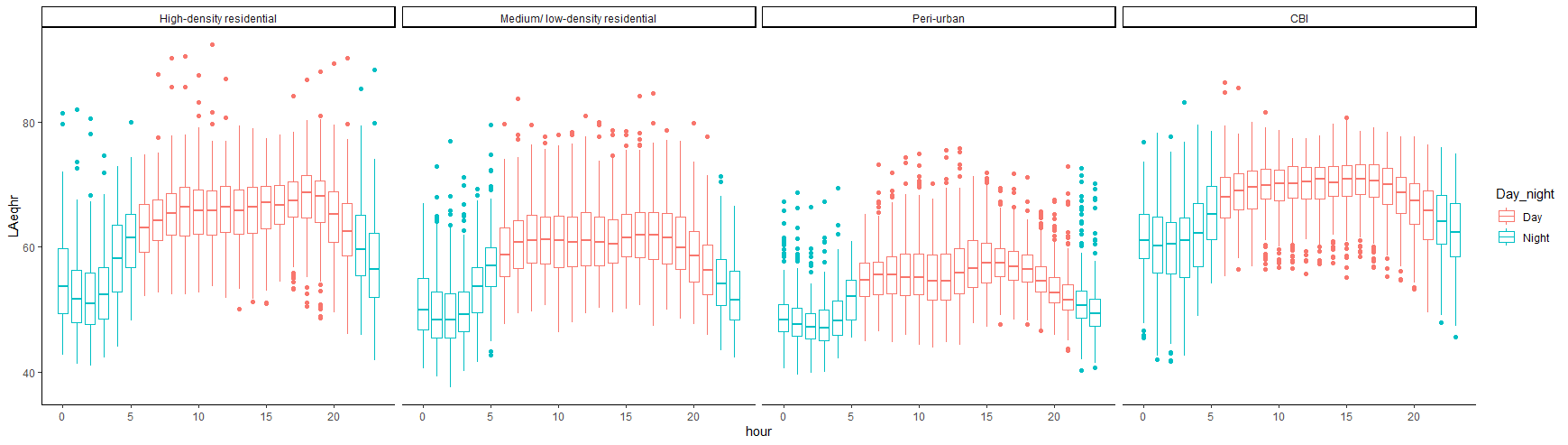
**

**Figure 3. Distribution of hourly measured sound levels (LAeq_1hr_) stratified by land use in the Greater Accra Metropolitan Area.** Upper and lower limits of the bounding boxes represent the interquartile range (IQR) and the horizontal line within the box the median. Outlying points on the figure represent outlier data. Data from 136 rotating sites but fixed sites had similar diurnal patterns.

**
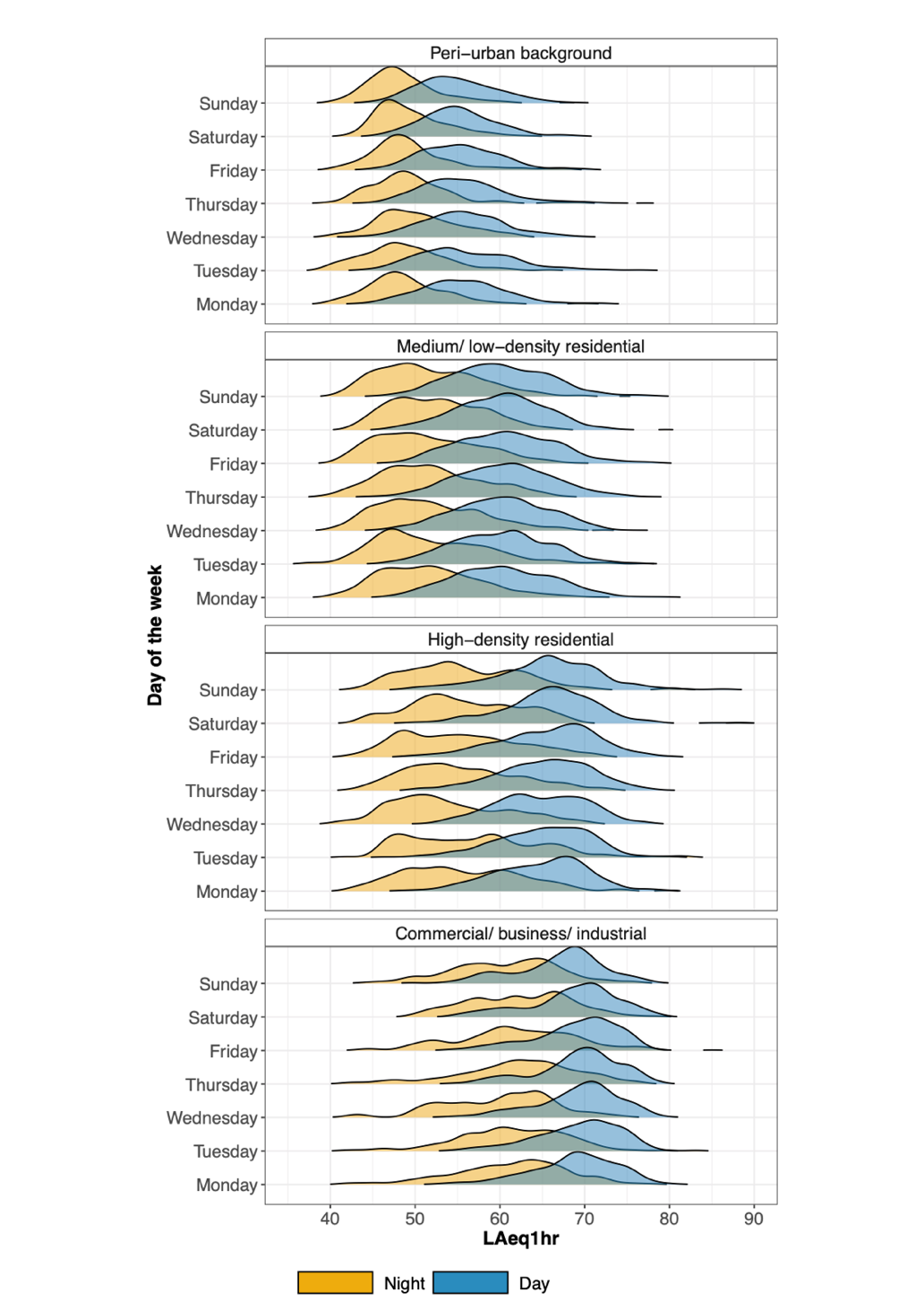
**

**Figure 4. Distribution of day and night-time hourly sound levels (LAeq_1hr_) across days of the week and land use areas among 136 rotating sites in the Greater Accra Metropolitan Area.** Weekday differences in medians for each land use area were not significant (p-value (p) range: 0.20 – 0.95).

**
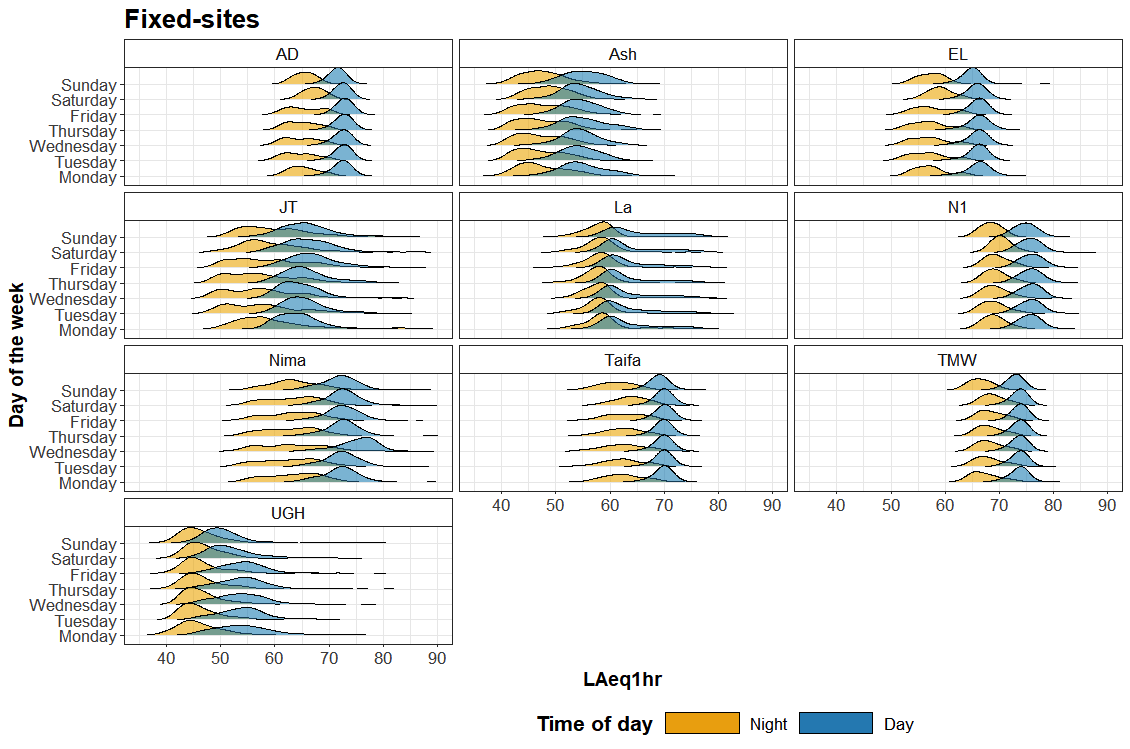
**

**Figure 5. Distribution of day and night-time hourly sound levels (LAeq_1hr_) across days of the week and each of the 10 fixed sites in the Greater Accra Metropolitan Area.** N1 West at Lapaz (**N1**) and Tema Motorway (**TMW**) are at the west and east ends of the multi-lane N1 motorway; Asylum Down (**AD**) is on the Ring Road Central; Jamestown (**JT**) and Nima (**Nima**) are poor, densely populated neighborhoods in south and middle of Accra Metropolis; Taifa (**Taifa**) is an emerging neighborhood north of the city; Labadi (**La**) is an indigenous Ga community along on the Coast; East Legon (**EL**) is an affluent neighborhood next to the University of Ghana Campus. Previously residential streets in EL now host large corporate, commercial and small business ventures; Ashaiman (**Ash**) is an emerging neighborhood next to the port city of Tema; and University of Ghana Hill (**UGH**) is located on top of the quiet, forested Legon Hill.

The majority of fixed sites had relatively consistent day and night-time measured hourly sound levels across different days of the week, though the medians on Sundays were different from other days (p<0.05) at sites influenced by road traffic (EL, TMW, AD, Taifa, N1) and at the University (UGH). At Nima, Wednesdays had higher sound levels in the daytime compared to other days (p<0.01). This is accounted for by the fact that Wednesdays at Nima are busy market days.


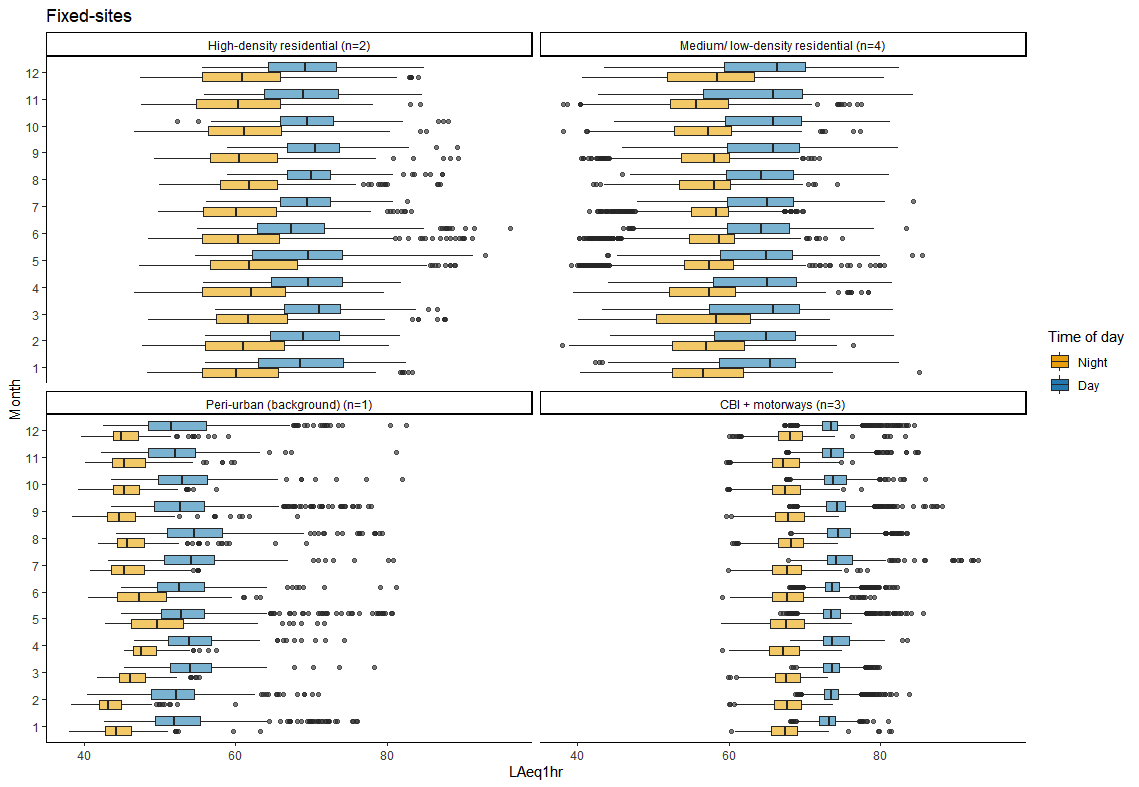


**Figure 6. Median and inter-quartile ranges (IQR) of day and night-time hourly sound levels (LAeq_1hr_) across months of the year stratified by land use category.** Data from 10 yearlong fixed sites. ‘n’ represents the number of sites in each land use category. Points on the figure represent outlier data.

The majority of land use areas had relatively consistent day and night-time measured hourly sound levels across different months of the year and the medians were not different (p>0.05). The least within and between month variation was observed at the commercial, business, and industrial (CBI) sites along major motorways (n=3). UGH is the peri-urban site at the top of University Hill at the University of Ghana. This is a secluded forested area with very few buildings and a one lane road. The increase of median sound levels from April – June compared to other months at the UGH site (p<0.05) is possibly due to University graduation and commencement activities which take place around this time of the year near the Administrative building on University Hill, close to where the monitors are located. We also observed that there were some additional construction/ landscaping activities taking place in this area during these months.


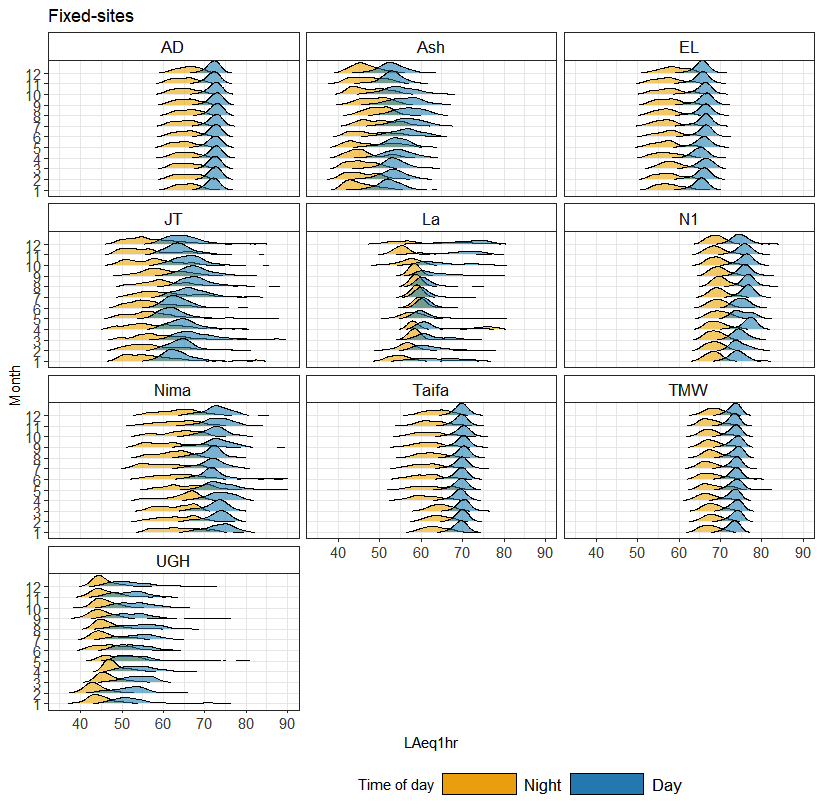


**Figure 7. Distribution of day and night-time hourly sound levels (LAeq_1hr_) across months of the year stratified by each of the 10-fixed sites.** N1 West at Lapaz (**N1**) and Tema Motorway (**TMW**) are at the west and east ends of the multi-lane N1 motorway; Asylum Down (**AD**) is on the Ring Road Central; Jamestown (**JT**) and Nima (**Nima**) are poor, densely populated neighborhoods in south and middle of Accra Metropolis; Taifa (**Taifa**) is an emerging neighborhood north of the city; Labadi (**La**) is an indigenous Ga community along on the Coast; East Legon (**EL**) is an affluent neighborhood next to the University of Ghana Campus. Previously residential streets in EL now host large corporate, commercial and small business ventures; Ashaiman (**Ash**) is an emerging neighborhood next to the port city of Tema; and University of Ghana Hill (**UGH**) is located on top of the quiet, forested Legon Hill.

The majority of land use areas had relatively consistent day and night-time measured hourly sound levels across different months of the year. The least within and between month variation was observed at the commercial, business, and industrial (CBI) sites along major motorways, including TMW, AD, N1, as well as the site at EL. The high-density residential sites (JT, Nima) appeared to have more within-month (e.g., day to day random variation) variability than between month variability. Day-time sound levels at the La site near the ocean varied throughout the year, likely due to a nearby resort which was undergoing periodic periods of construction and renovation. Discussion on monthly sound levels at UGH can be found with Figure 6.

**S3. Measured sound levels prior to and during the *Homowo* festival in Accra**

**Table 2. Sound levels during and before/after the one month ban on noise making in Accra.** Data are summarized as medians and inter-quartile ranges (IQR) of hourly sound levels (LAeq_1hr_) at 10 fixed-site locations. In Accra a ban on drumming and noise making for 1 month prior to the *Homowo* festival is mandated by the traditional and indigenous authorities in Accra (May-13^th^ – June 13^th^ 2019).

|  | High-density residential  (n=2 sites) | Medium/low-density residential  (n=4 sites) | Peri-urban (background)  (n=1 site) | Commercial, business, industrial (n=3 sites) |
| --- | --- | --- | --- | --- |
| One month prior to and one month after the noise ban | 66.8 (62.5, 71.7) dBA | 61.4 (57.2, 66.9) | 51.4 (48.1, 54.9) | 73.1 (69.1, 74.6) |
| During the one-month noise ban | 64.1 (60.1, 72.0) dBA | 60.4 (56.7, 66.6) | 51.5 (48.3, 55.0) | 72.7 (70.0, 74.8) |

**S4. Prevalence of sound sources across days of the week and months of the year**


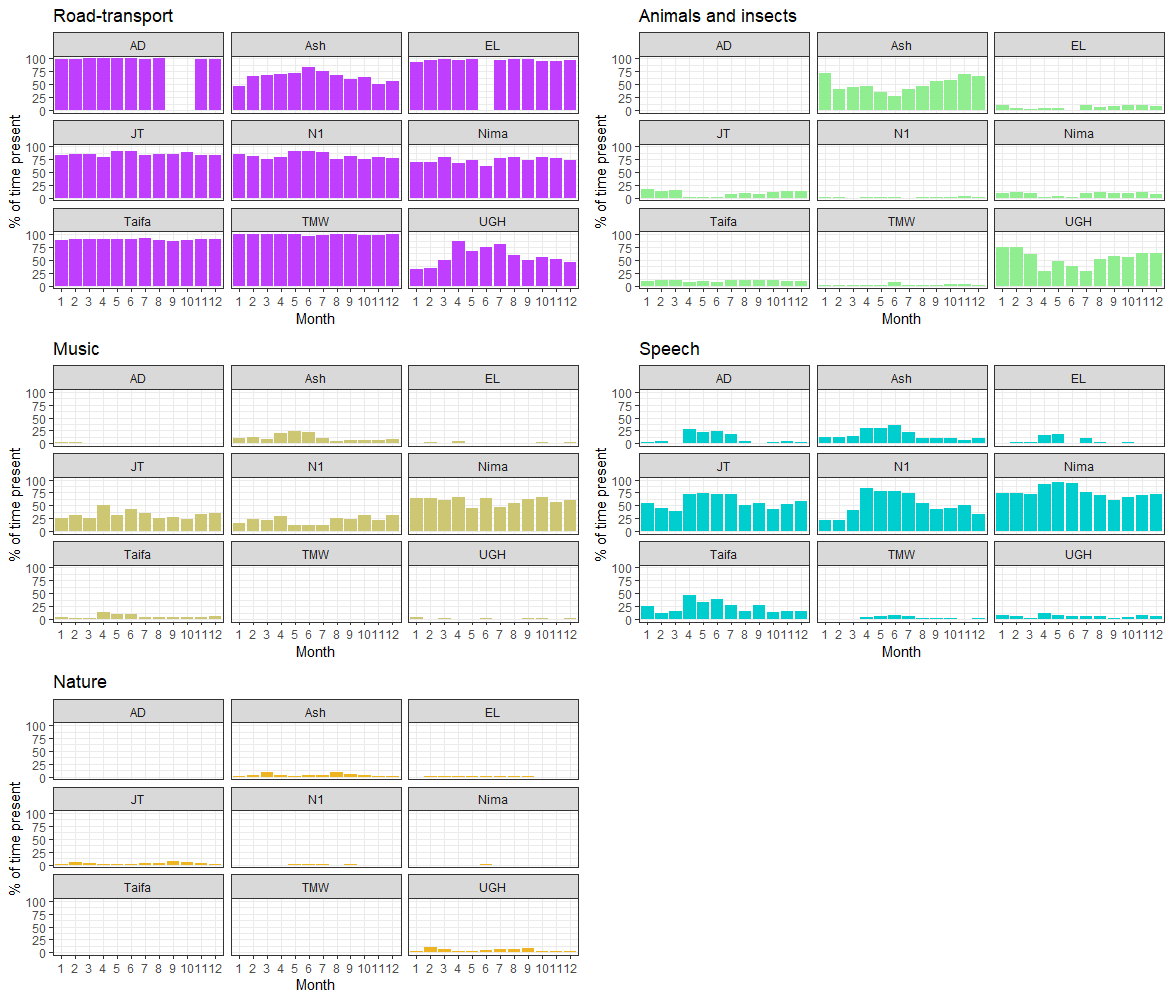


**Figure 8. Sound source prevalence (% of time present) across months of the year for each fixed site.** N1 West at Lapaz (**N1**) and Tema Motorway (**TMW**) are at the west and east ends of the multi-lane N1 motorway; Asylum Down (**AD**) is on the Ring Road Central; Jamestown (**JT**) and Nima (**Nima**) are poor, densely populated neighborhoods in south and middle of Accra Metropolis; Taifa (**Taifa**) is an emerging neighborhood north of the city; Labadi (**La**) is an indigenous Ga community along on the Coast; East Legon (**EL**) is an affluent neighborhood next to the University of Ghana Campus. Previously residential streets in EL now host large corporate, commercial and small business ventures; Ashaiman (**Ash**) is an emerging neighborhood next to the port city of Tema; and University of Ghana Hill (**UGH**) is located on top of the quiet, forested Legon Hill. Note that the AD site was missing 2 months of data (September – October) and EL (July) one month of data due to equipment shortages.

Prevalence of road-transport sounds was fairly constant at fixed sites throughout the year, though UGH site appeared to have a higher prevalence during the months of April – July. Similarly, animal and insect sound during these months at this site were lower than other months, possibly as a result of animals avoiding vehicles or a potential masking effect of louder road-transport sounds in the audio recordings. There wasn’t a clear seasonal pattern of music sounds, though some minimal month-to-month random variation. The prevalence of speech sounds was noticeably higher at Nima, N1, JT, Taifa, Ash, AD, and EL between April and July. There was little month-to-month variability in nature sounds.


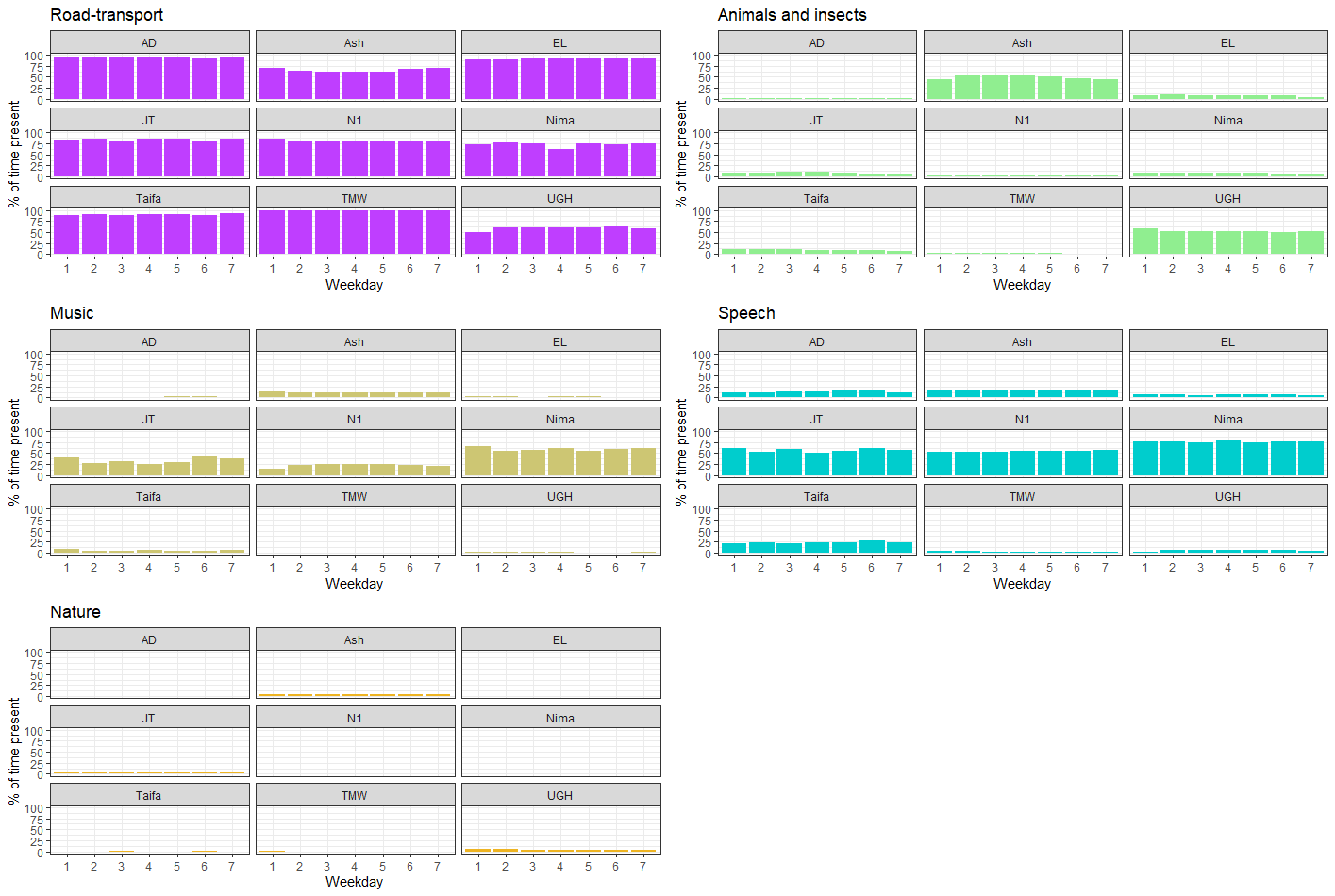


**Figure 8. Sound source prevalence (% of time present) across days of the week for each fixed site.** N1 West at Lapaz (**N1**) and Tema Motorway (**TMW**) are at the west and east ends of the multi-lane N1 motorway; Asylum Down (**AD**) is on the Ring Road Central; Jamestown (**JT**) and Nima (**Nima**) are poor, densely populated neighborhoods in south and middle of Accra Metropolis; Taifa (**Taifa**) is an emerging neighborhood north of the city; Labadi (**La**) is an indigenous Ga community along on the Coast; East Legon (**EL**) is an affluent neighborhood next to the University of Ghana Campus. Previously residential streets in EL now host large corporate, commercial and small business ventures; Ashaiman (**Ash**) is an emerging neighborhood next to the port city of Tema; and University of Ghana Hill (**UGH**) is located on top of the quiet, forested Legon Hill. Sunday **(1)**, Monday **(2)**, Tuesday **(3)**, Wednesday **(4)**, Thursday **(5)**, Friday **(6)**, Saturday **(7)**.

Fixed sites had relatively consistent prevalence of sound sources across different days of the week. Though, the prevalence of music sounds were slightly higher on Sundays, particularly in the high-density residential areas (Nima, JT) and in Taifa.

**S5. Short-term representativeness of long-term sound levels**

**Table 3. The difference between randomly sampled short-term (daily) sound levels (LAeq_24hr_) and year-long averages across 10-fixed site locations.** Each site had 5 random days sampled from its entire distribution of available days. The short-term data for each site were compared to the site-specific yearly average.

| Land use classification | Site ID | Median difference (dBA) |
| --- | --- | --- |
| Commercial, business, industrial (also along major road/ highway) |  |  |
|  | N1 | -0.63 |
|  | TMW | -0.16 |
|  | AD | 0.19 |
| High-density residential |  |  |
|  | Nima | -0.58 |
|  | JT | 2.59 |
| Medium/ low-density residential |  |  |
|  | Taifa | -0.15 |
|  | Ash | -1.17 |
|  | La | -1.32 |
|  | EL | 0.71 |
| Peri-urban |  |  |
|  | UGH | -2.07 |

N1 West at Lapaz (**N1**) and Tema Motorway (**TMW**) are at the west and east ends of the multi-lane N1 motorway; Asylum Down (**AD**) is on the Ring Road Central; Jamestown (**JT**) and Nima (**NM**) are poor, densely populated neighborhoods in south and middle of Accra Metropolis; Taifa (**TF**) is an emerging neighborhood north of the city; Labadi (**La**) is an indigenous Ga community along on the Coast; East Legon (**EL**) is an affluent neighborhood next to the University of Ghana Campus. Previously residential streets in EL now host large corporate, commercial and small business ventures; Ashaiman (**Ash**) is an emerging neighborhood next to the port city of Tema; and University of Ghana Hill (**UGH**) is located on top of the quiet, forested Legon Hill.

**S6. The percentage of site-days in the Greater Accra Metropolitan Area where measured day-time (L_day_) and night-time (L_night_) sound levels at residential and mixed-used areas exceeded Ghana Standards Authority permissible limits for**

**environmental sound.**

**Table 4. Ghana Standards Authority permissible limits for environmental sound** ^2^**.**

| Zones defined by the Ghana Standards Authority | **Permissible L_day_, L_night_ limits*** |
| --- | --- |
| Residential areas | **55, 48 dBA** |
| Mixed use areas | **60, 55 dBA** |

*L_day_: covers daytime hours from 6:00am to 9:59pm; L_night_: covers nighttime hours from 10:00pm to 5:59am.

Our land use categorizations do not fully match the zones described by the Ghana Standards Authority. However, we compared our land use areas to the most comparable zones to provide results that are indicative and general, and the comparison is not intended as a formal compliance evaluation for these areas. We also made these comparisons for our rotating sites only.

We compared our peri-urban residential sites to the Ghana Standards Authority ‘Residential area’ zones. Under this comparison, we found that 79% of site-days of day-time sound levels (L_day_) surpassed the guideline limit. As well, 83% of site-days of night-time sound levels (L_night_) surpassed the limit. Our peri-urban sites have the lowest density of built-up areas, lowest population density, and the most vegetation compared to our other land use areas.

We compared our medium/low-density residential areas to the Ghana Standards Authority ‘Residential area’ zones. Under this comparison, we found that 95% of site-days of day-time sound levels (L_day_) surpassed the guideline limit. As well, 94% of site-days of night-time sound levels (L_night_) surpassed the limit.

We compared our high-density residential areas to the Ghana Standards Authority ‘Residential area’ zones and ‘Mixed area’ zones as these areas are typically both residential and contain a high density of mixed activities. Under this comparison, we found that 99% of site-days of both day-time and night-time sound levels surpassed the guideline limit for ‘Residential areas’. As well, 93% of site-days of day-time sound levels and 71% of night-time sound levels surpassed the limits set for ‘Mixed use areas’.

**S7. Pre, during, and post COVID lockdown measured sound levels at fixed sites.**


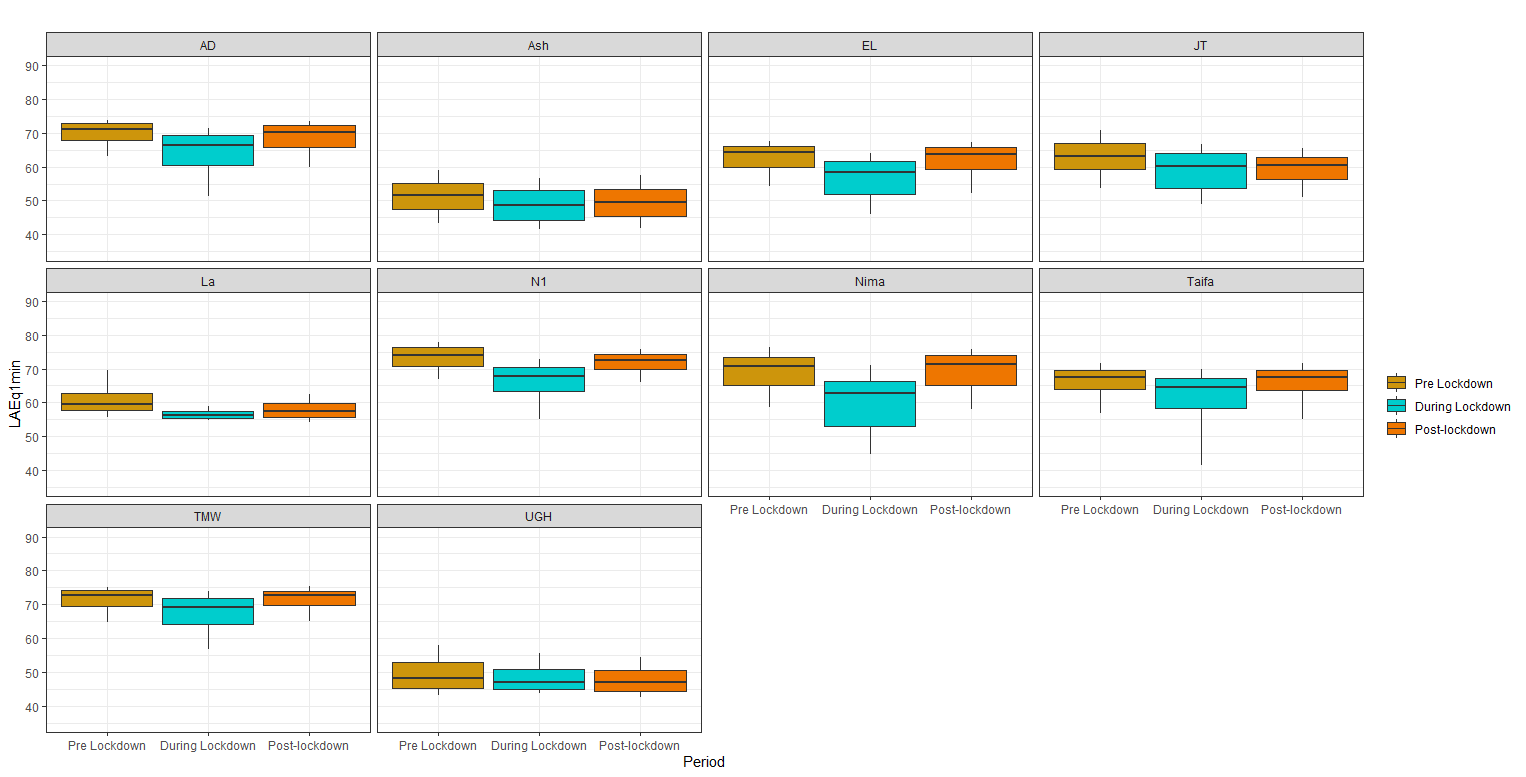


**Figure 10. Distribution of pre-, during, and post-COVID-19 lockdown 1-minute sound levels at 10-fixed sites in the GAMA.** Box and whisker plots show the 10^th^ and 90^th^ percentiles as the upper and lower limits of the vertical black lines, the 25^th^ and 75^th^ percentiles (IQR) as the upper and lower limits of the box and the median as the horizontal line within the box. With the exception of JT and La, post-lockdown sound levels returned to pre-lockdown levels. Lockdown period covered one week with measurements from March 30^th^ – April 8^th^. Post-lockdown period was from May 15^th^ 2020 to June 15^th^ 2020. N1 West at Lapaz (**N1**) and Tema Motorway (**TMW**) are at the west and east ends of the multi-lane N1 motorway; Asylum Down (**AD**) is on the Ring Road Central; Jamestown (**JT**) and Nima (**Nima**) are poor, densely populated neighborhoods in south and middle of Accra Metropolis; Taifa (**Taifa**) is an emerging neighborhood north of the city; Labadi (**La**) is an indigenous Ga community along on the Coast; East Legon (**EL**) is an affluent neighborhood next to the University of Ghana Campus. Previously residential streets in EL now host large corporate, commercial and small business ventures; Ashaiman (**Ash**) is an emerging neighborhood next to the port city of Tema; and University of Ghana Hill (**UGH**) is located on top of the quiet, forested Legon Hill.

Data on noise levels in Accra during the COVID lockdown period were from one week of measurement when we could continue some fieldwork without compromising safety and movement restrictions (the field team left the equipment to run for as long as it could, but then they were not permitted to visit sites to swap equipment after 7-days (as per our usual schedule)). Reductions in this week were lower than what was observed in Barcelona (~9-11 dBA decrease in L_day_) ^3^ and Lima (~8 dBA decrease in L_den_) ^4^ but similar to London (5.4 dBA decrease in LAeq (range 1.2 to 10.7 dBA)) ^5^. Across the 10-fixed sites, pre-lockdown median LAeq_1min_ was 65 dBA, while during lockdown it was 59 dBA, representing a difference of ~6 dBA. Though, these differences varied between sites (range: 1 dBA to 7 dBA difference in median LAeq_1min_). Differences could be due to different lockdown measures applied in each city as well as enforcement and adherence, especially in a setting where opportunity for remove work is limited.

**S8. Site-type (land use) classifications**

**Table 5. Land use classification descriptions and images.** Images from Google Street View, Google Earth, and our Accra campaign cameras.

| **Land use area** | **Descriptions** | **Street view and arial images** | |
| --- | --- | --- | --- |
| **Peri-urban ‘background’ areas** | Areas with an abundance of forest, grass land, shrubs, barren lands, water, and/or formal parkland.  Unpaved or paved roads with very low traffic volume. If residential homes exist, there are few.  *Example areas: University of Ghana hill or the Water Research Institute.* | **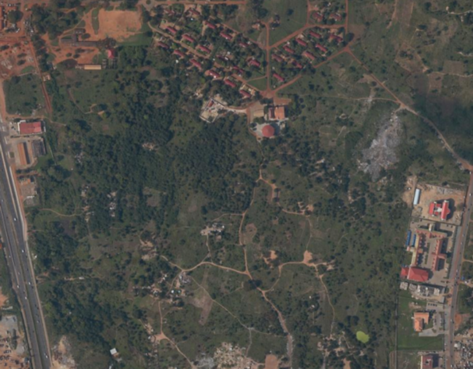**  **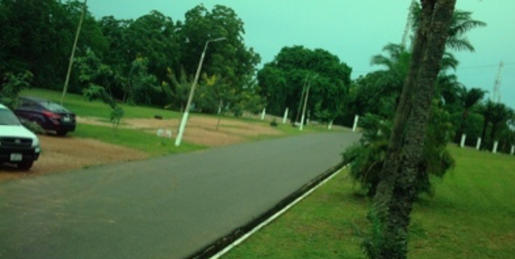** | 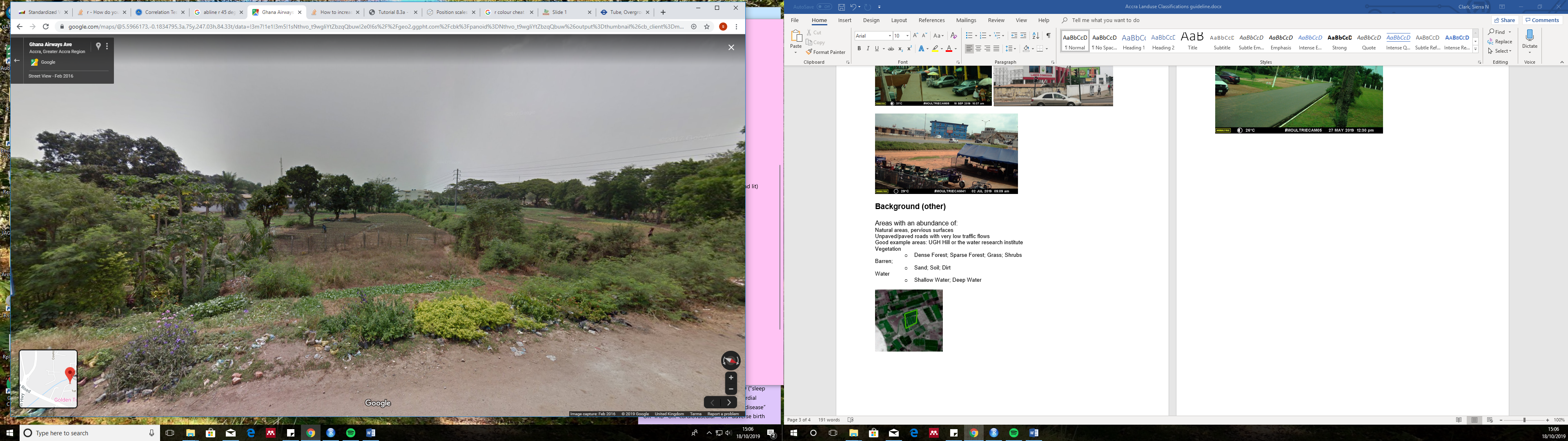  **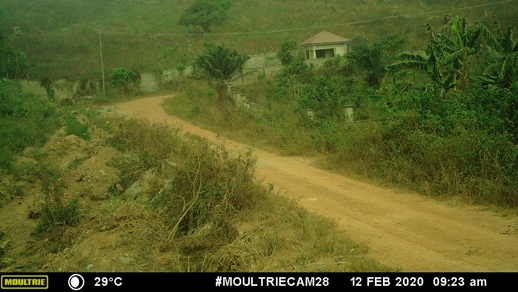** |
| **Medium/ low-density residential areas** | Formal residential areas. Medium to wide roads. Paved or unpaved and double or single lane roads. Houses can have yards, fences, walls, and/or driveways. Clear demarcation of where one home ends and another begins. Houses can be sparse or close together. Mostly residential buildings.  Population density (# people/room) is low (e.g., <2).  *Example locations: East Legon, Cantonments, Taifa.* | **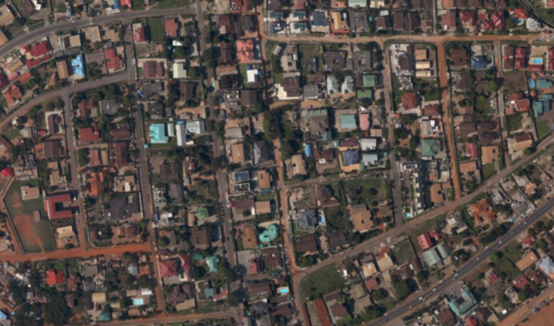**  **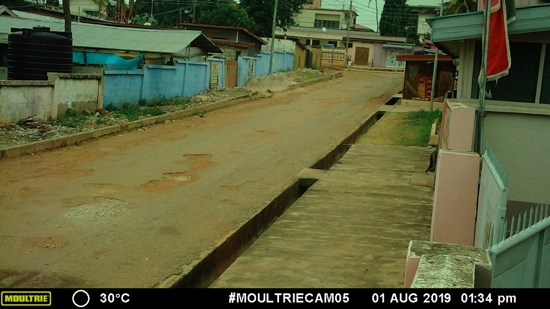** | 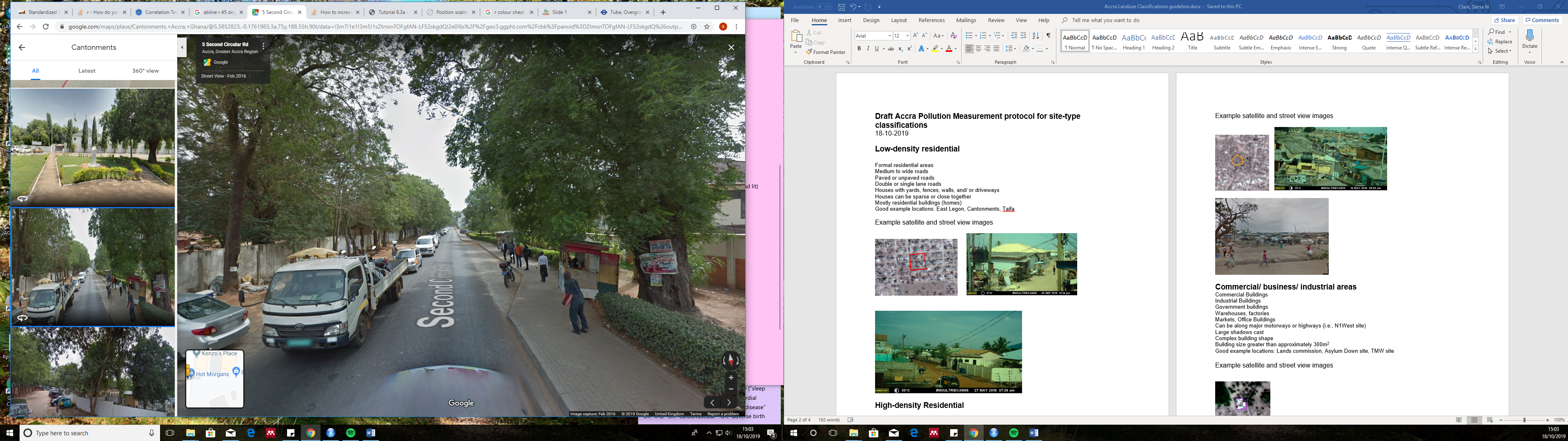  **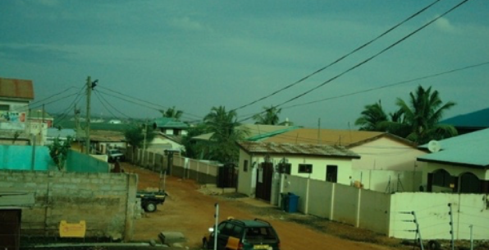** |
| **High-density residential areas** | Typically informal residential. Can be shantytowns/ slums. High population density (# people/ rooms, e.g., >2 people per room). Narrow, sometimes unidentifiable, paved or unpaved roads. Small building structures. No clear demarcation of where one home ends and another begins.  *Example locations: Jamestown, Nima, Old Fadama.* | **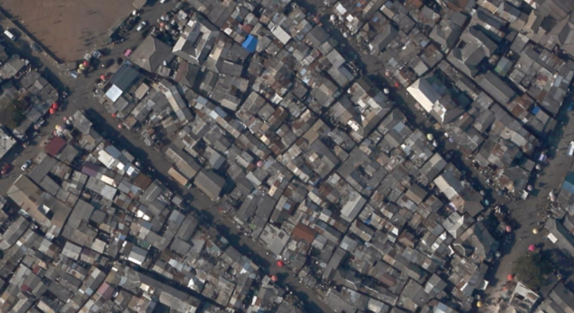**  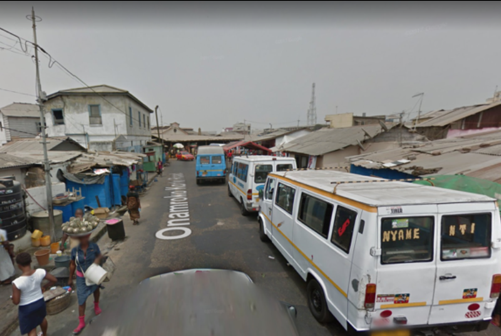 | 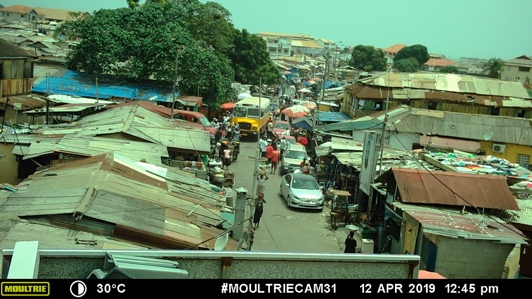 |
| **Commercial, business, and industrial areas** | Can be commercial buildings, industrial buildings, government buildings, warehouses, factories, markets, and/or office buildings. Can be along major motorways or highways (i.e., N1 motorway going west).  Generally large buildings.  *Example locations: Lands Commission.* | **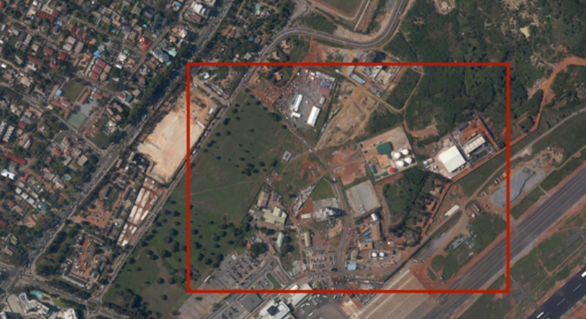**  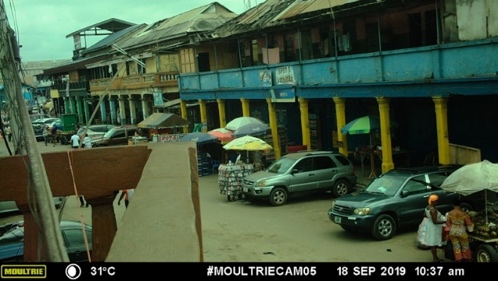 | 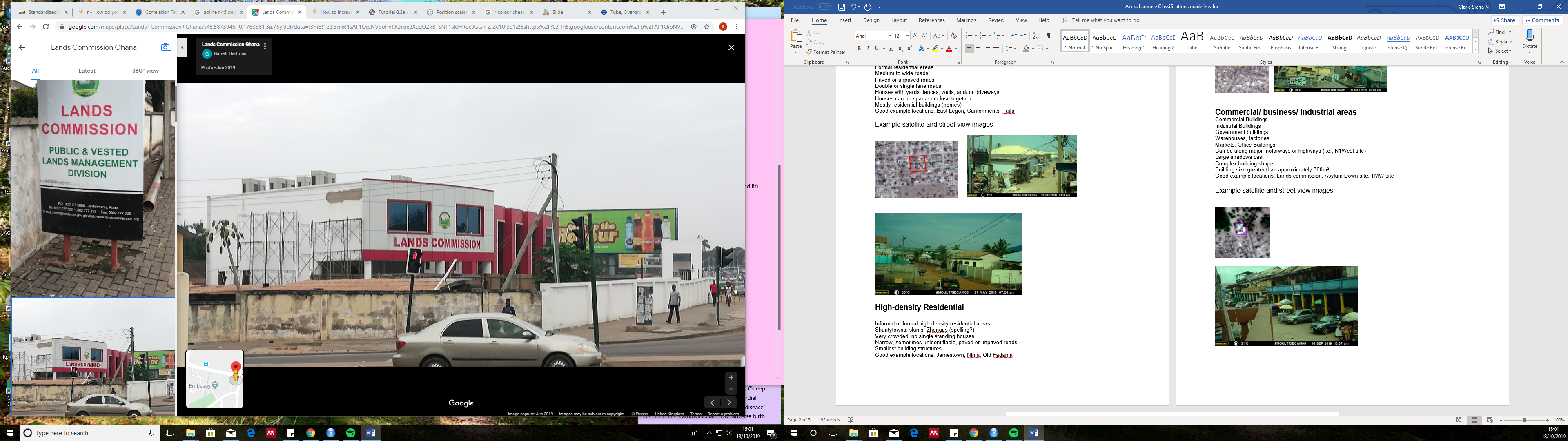  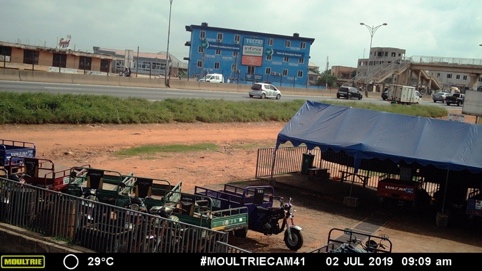  **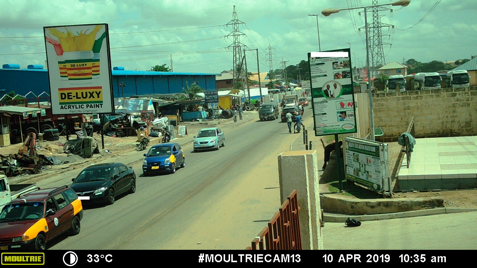** |

**S9. Quality assurance/ quality control**

Throughout the campaign, we looked for drift between the Noise Sentry sound level meters by collecting duplicate measurements at 16% of the rotating sites. We found that the median and mean difference between 1-minute duplicate measurements was -0.1 dBA and -0.04 dBA, respectively. Furthermore, the median and mean absolute differences between duplicate measurements was 0.74 dBA and 1.27 dBA. Figure 11 shows correlation between duplicate measurements when energy averages were summarized daily (LAeq_24hr_). Additional details on QA/QC can be found in our protocol paper ^6^.


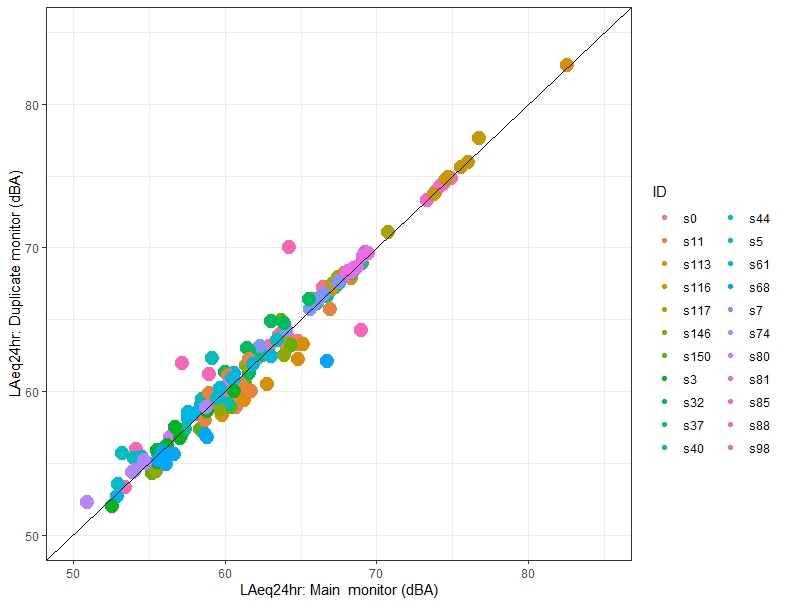


**Figure 11. Scatterplot of the correlation between the daily LAeq_24hr_ from the main and duplicate monitors conducted at 16% of rotating sites.** ID: measurement site identifiers. Note that site s88 was found to have faulty monitor.

**S10. Equation for Intermittency Ratio (IR) calculation**

IR equation re-created from Wunderli et al. 2016 ^1^

$$L_{eq,T,Tot}=10*log10(\frac{1}{T}\int_{0}^{T} {10}^{0.1L\left( t \right)}dt)[dB]$$

$$K=L_{eq,T,Tot}+C[dB]$$

$$L_{eq,T,Events}=10*Log10(\frac{1}{T}\int_{0}^{T} H\left( L\left( t \right)-K \right){10}^{0.1L\left( t \right)}dt)[dB]$$

$$IR=\frac{{10}^{0.1L_{eq,T,Events}}}{{10}^{0.1L_{eq,T,Tot}}}*100$$

*IR :* Intermittency Ratio

*L_eq,T,Tot_* : Overall sound pressure level for time T

*L_eq,T,Events_* : Event-based sound pressure level for time T

*L(t)* : Continuous sound pressure level at the receiver position

*K* : Threshold for which L(t) would contribute to L_eq,T,Events_

C : Fixed offset above L_eq,T,tot_

H: Heaviside step function

The free parameter C was set to +3 dBA above the L_eq,T,tot_ for our study based on earlier work that had been done ^1^ which showed that C=3 resulted in a fairly uniform spread of IR across a range of exposure situations. We also calculated and reported IRs from our Accra data with C = 4 and C = 5. IR’s that were calculated with C=3, C=4, and C=5 were correlated (C(3) and C(4): r=0.99; C(3) and C(5): r=0.95) across measurement sites and days, though the absolute values of IRs were lower when C=5 (S1).

**S11. Sound source data cleaning and model testing**

We applied a pre-trained neural network attention model (DEEP-Hybrid DataCloud project ^7^ based on ^8^), to our audio recordings to classify the different types of sounds present in the audio. After an audio file was passed through the model, we retained the model’s top 3 returned sound classes. The model sometimes returned fine-grained predictions, such as ‘owl’ instead of a higher order grouping such as bird or animal, resulting in hundreds of different types of sound classes returned. For interpretability and to be in line with acoustic environment classifications ^9^, we grouped the model’s fine-grained predictions into higher-order categories representing sound sources. These grouped categories include, road-transport sounds, animal and insect sounds, outdoor music, human speech, static/ buzz, airplane sounds, nature sounds (e.g., rain, thunder) and miscellaneous sounds which did not fit into any of the aforementioned sound source categories. For simplicity during analysis, we grouped miscellaneous and static/ buzz into a category called ‘other’.

The accuracy of the model predictions in their sound source categories from the Accra campaign data was tested for agreement against researcher labeled audio recordings. We randomly selected 150, 10-second audio clips (1/3 from rotating sites, 2/3 from fixed sites) that were representative of all times of the day and the entire measurement campaign, and manually labelled (blinded to the results) the sound classes/types observed in the files. Both the researcher labels and model predictions were then grouped into the higher-order sound source categories. The accuracy, positive predictive value (PPV), and negative predictive value (NPV) for each sound source is reported.

Generally,, road-transport, animal and insect, outdoor music, and human speech sounds had moderate-high to high PPV. The model did not predict nature sounds often, but when it did, it was with high accuracy (PPV: 100%, NPV: 83%). The model preformed poor with airplane sounds, which were only labelled as occurring once by the researcher but predicted as present by the model 26 times.

**Table 6. Acoustic classifier model accuracy, positive predictive values, and negative predictive values when applied to the Accra campaign data.**

| Sound source category | Accuracy | Positive predictive value | Negative predictive value |
| --- | --- | --- | --- |
| Road-transport | 76% | 76% (n=93/ 123) | 78% (n=21/27) |
| Animals and insects | 79% | 97% (n=30/31) | 74% (n=88/119) |
| Music | 93% | 71% (n=18/25) | 97% (n=121/125) |
| Speech | 80% | 83% (n=39/47) | 79% (n=81/103) |
| Airplane | 82% | 4% (n=1/26) | 99% (n=123/124) |
| Nature | 87% | 100% (n=4/4) | 83% (n=126/146) |

%: percentage; n: number of audio files

**Accuracy**: (True positive + True negative) / Total observations

**Positive predictive value**: True positive/ (True positive + False positive): Percentage of audio files where the sound source was labelled as present by the researcher given that it was predicted as present by the model.

**Negative predictive value**: True negative/ (True negative + False negative): Percentage of audio files where the sound source was labelled as not present by the researcher given that it was predicted not to be present by the model.

We believe the reason that the neural network was poor at identifying airplane sounds within our audio dataset was because we wanted to characterise the complexity of the city, and aircraft traffic is limited to one area. So rather than putting a lot of monitors on flight paths – which one would do where there are major policy decisions for airports – we prioritised everywhere. Thus, if we did record a sound of an airplane, we believe it would have been quite faint, and difficult to distinguish amongst other, more proximal, sounds picked up in the audio. This would then lead to either a masking effect or the neural network not being able to distinguish the airplane sound as an airplane.

**S12. Distribution of 1-minute sound levels (LAeq_1min_) among data used to create Figure 3 in the main paper.**

**
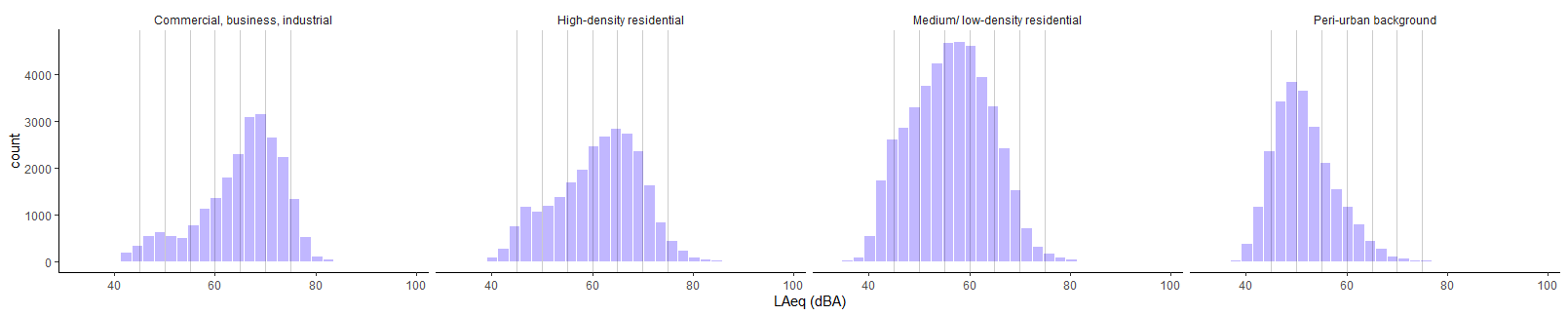
**

**Figure 12. Distribution of 1-minute sound levels used to create Figure 3 in the main paper.** The vertical grey lines represent the positions of the LAeq_1min_ intervals in Figure 3.

We collected audio recordings every 10-minutes, but continuously recorded sound levels throughout the campaign every minute. Thus, the sound level dataset used to create Figure 3 and represented in the above histogram is 1/10^th^ of the size of the full sound level dataset.

**References**

1. Wunderli, J. M. *et al.* Intermittency ratio: A metric reflecting short-term temporal variations of transportation noise exposure. *J. Expo. Sci. Environ. Epidemiol.* **26**, 575–585 (2016).

2. Ghana Standards Authority. *Health protection - requirements for ambient noise control*. (2018) doi:GS 1222:2018.

3. Barcelona, A. de. Informe COVID-19 d’alteració dels nivells sonors. https://ajuntament.barcelona.cat/%0Aecologiaurbana/ca/serveis/la-ciutat-funciona/mantenimentde-l-espai-public/gestio-energetica-de-la-ciutat/servei-decontrol-acustic/informe-covid-19 (2020).

4. Montano, W. & Gushiken, E. Lima soundscape before confinement and during curfew. Airplane flights suppressions because of Peruvian lockdown. *J. Acoust. Soc. Am.* **148**, 1824–1830 (2020).

5. Aletta, F., Oberman, T., Mitchell, A., Tong, H. & Kang, J. Assessing the changing urban sound environment during the COVID-19 lockdown period using short-term acoustic measurements. *Noise Mapp.* **7**, 123–134 (2020).

6. Clark, S. *et al.* High-resolution spatiotemporal measurement of air and environmental noise pollution in sub-saharan african cities: Pathways to Equitable Healthy Cities Study protocol for Accra, Ghana. *BMJ Open* 1–10 (2020) doi:http://dx.doi.org/10.1136/bmjopen-2019-035798.

7. Lopez Garcia, A. *et al.* A cloud-based framework for machine learning workloads and applications. *IEEE Access* **8**, 18681–18692 (2020).

8. Changsong, Y., Barsim, K. S., Kong, Q. & Yang, B. Multi-level attention model for weakly supervised audio classification. *arXiv Prepr. arXiv* (2018) doi:1803.02353.

9. ISO. *PD ISO / TS 21830 : 2018 Acoustics - Soundscape: Part 2: Data collection and reporting requirements*. (2018).
